# Strigolactones in Plant Abiotic Stress Resilience: Hormonal Crosstalk, Mechanistic Regulation, and Agricultural Prospects

**DOI:** 10.3390/plants15121855

**Published:** 2026-06-15

**Authors:** Cheng Huang, Lin Wu, Jia Xiong, Hua Liu, Yuhua Ma, Xumei Luo, Leiru Chen, Fasih Ullah Haider, Yan Chen

**Affiliations:** 1 Hubei Key Laboratory of Biologic Resources Protection and Utilization, Hubei Minzu University, Enshi 445000, China; 2024102@hbmzu.edu.cn (C.H.); 2015063@hbmzu.edu.cn (L.W.); 2024125@hbmzu.edu.cn (J.X.); 202440495@hbmzu.edu.cn (Y.C.); 2 Anhui Provincial Key Laboratory of Forest Resources and Silviculture, Anhui Agricultural University, Hefei 230036, China; liuhuanmg@ahau.edu.cn; 3 College of Civil and Architecture Engineering, Chuzhou University, Chuzhou 239099, China; myh@chzu.edu.cn; 4 Anhui Academy of Forestry, Hefei 230031, China; 5 South China Botanical Garden, Chinese Academy of Sciences, Guangzhou 510650, China; haider281@scbg.ac.cn

**Keywords:** abiotic stress, antioxidant defense, climate-resilient agriculture, hormonal crosstalk, stress resilience, strigolactones

## Abstract

Strigolactones (SLs) have emerged as important regulators of plant adaptation to abiotic stress, functioning not as isolated hormones but as integrative signaling molecules. Beyond stress responses, SLs regulate key biological processes, including shoot branching, root architecture, leaf senescence, nutrient acquisition, rhizosphere communication, flowering-related development, and growth–developmental plasticity. This review synthesizes current knowledge on how SLs modulate plant responses to drought, salinity, heavy metal toxicity, high temperature, and low temperature through crosstalk with abscisic acid, auxin, cytokinin, ethylene, and gibberellin. We examine SL structural diversity, biosynthesis, transport, and signaling together with their roles in growth–stress coordination, hormonal networking, and stress-specific mitigation, while distinguishing endogenous SL functions from responses inferred from exogenous analogs such as GR24. Across stresses, SL-mediated resilience converges on adaptive modules, including water regulation, root–shoot architectural remodeling, redox protection, ion and osmotic homeostasis, photosynthetic maintenance, and rhizosphere-assisted resource acquisition. The mechanistic basis involves transcriptional reprogramming, ROS/RNS-linked redox regulation, metabolic protection, and root–microbe interactions. Translational prospects include SL analogs, genetic manipulation, and breeding for adaptive plasticity, nutrient efficiency, and stress tolerance. However, species specificity, dosage dependence, limited field validation, unclear structure–function relationships, and parasitic-weed stimulation remain major constraints.

## 1. Introduction

Abiotic stress is now recognized as one of the most serious threats to agricultural productivity and food security worldwide [1]. Among the major abiotic constraints affecting crop production are salinity, drought, heavy metal toxicity, high temperature, and low temperature. These are particularly important due to their broad occurrence and direct effects on crop growth and yield formation [2]. Salinity alone affects about 20–23% of cultivated land and 25–33% of irrigated land worldwide. This poses a major constraint to agricultural sustainability and crop productivity [3,4]. Temperature stress is also increasingly important with climate change. For instance, a 1 °C rise in global mean temperature has been linked to mean yield reductions of about 6% in wheat (*Triticum aestivum* L.), 3.2% in rice (*Oryza sativa* L.), 3.1% in soybean (*Glycine max* L.), and 7.4% in maize (*Zea mays* L.). In some analyses, yield losses due to heat stress alone reach 40% [4]. Recent syntheses estimate that abiotic stress accounts for 51–82% of annual global crop yield losses. Wider agronomic assessments indicate that nearly 90% of cultivable land is affected by adverse abiotic factors. Under severe exposure, the yield of major food crops may be reduced by up to 70% [1,2]. These stresses are destructive because they converge on sensitive cellular and physiological targets [5]. Drought mainly restricts tissue hydration, stomatal conductance, and carbon assimilation. Salinity causes both osmotic stress and ion toxicity. Heavy metals disrupt nutrient balance, membrane integrity, and redox homeostasis. Heat accelerates protein denaturation, membrane fluidization, and oxidative injury. Cold suppresses membrane function and photosynthetic metabolism, hinders growth, and increases membrane leakage and pigment loss [1,5]. Abiotic stresses lead to excessive formation of reactive oxygen species (ROS). This intensifies lipid peroxidation, chlorophyll degradation, enzyme dysfunction, and photosynthetic decline [6]. These effects are visible in crop plants. In maize, salinity reduces cob length, diameter, number of grains, total grain weight per cob, and 100-grain weight [5]. In tomato (*Solanum lycopersicum* L.), low-light stress decreases stem diameter, total fresh and dry weights, and chlorophyll *a* and *b* contents [5]. Under cadmium (Cd) stress, switchgrass (*Panicum virgatum* L.) and barley (*Hordeum vulgare* L.) show declines in chlorophyll content, photosynthetic rate, stomatal conductance, and transpiration [6,7]. Drought sharply reduces survival in *Arabidopsis thaliana*. Meanwhile, cold and heat stress increase electrolyte leakage, malondialdehyde (MDA) accumulation, oxidative damage, and photosynthetic dysfunction in sensitive plant species [5]. These abiotic stresses are increasing in frequency, intensity, and co-occurrence due to climate instability. Mitigating their damage is essential for maintaining yield stability and achieving sustainable crop production.

A wide range of agronomic and crop-management interventions has been employed to reduce abiotic stress damage, including irrigation scheduling, nutrient management, microbial inoculation, exogenous protectants, and conventional chemical growth regulation [8,9]. These approaches can undoubtedly provide short-term benefits, but in most cases, they remain input-dependent, context-sensitive, and variably reproducible across environments, particularly when stress intensity, soil conditions, genotype, and field management differ substantially [8,10]. In low-input agricultural systems, the repeated use of external amendments is often difficult to sustain economically, whereas in high-input systems, excessive dependence on fertilizers and pesticides may aggravate soil degradation, reduce environmental quality, and increase greenhouse gas emissions, thereby weakening the long-term sustainability of crop production [8]. For this reason, current plant-stress biology increasingly favors endogenous regulatory systems that enable plants to perceive stress, coordinate internal signaling, and reprogramme growth and metabolism more efficiently than purely input-based mitigation strategies [9,10,11]. Among these systems, phytohormones are especially compelling because they act at very low concentrations, yet exert disproportionately large effects on stress acclimation by integrating environmental cues with developmental regulation, nutrient partitioning, metabolic adjustment, and organ-level plasticity [9,10]. Rather than functioning as isolated signals, plant hormones operate through interconnected regulatory networks that coordinate molecular, physiological, biochemical, and morphological responses under adverse environments [11,12,13]. Therefore, a hormone-centered framework is attractive not simply because hormones are important regulators, but because it offers a mechanistically coherent and potentially more sustainable route by which stress perception can be translated into growth adjustment, redox buffering, resource reallocation, and survival under abiotic stress.

Within this regulatory framework, strigolactones (SLs) have emerged as one of the most distinctive signaling systems in plant stress biology [14]. SLs were first identified in 1966 as root exudates that stimulate germination of parasitic weeds such as *Striga*. Thus, they were initially associated with crop susceptibility rather than crop protection [15]. Their biological significance changed fundamentally when they were shown to stimulate hyphal branching in arbuscular mycorrhizal fungi (AMF). This revealed an essential role in rhizosphere communication and mineral acquisition [16]. A second conceptual advance came in 2008, when genetic and physiological studies established SLs as endogenous hormones that suppress shoot branching. This moved them from the category of external rhizosphere cues into the core of plant developmental regulation [17,18]. Since then, more than 30 natural SLs have been identified. Accumulating evidence has shown that they regulate root architecture, root hair elongation, secondary growth, leaf senescence, nutrient foraging, and stress-associated plasticity [19,20,21]. Their contribution to abiotic stress adaptation extends far beyond developmental control. Under stress, SLs improve plant performance through several recurring adaptive modules, including photosynthetic maintenance, antioxidant protection, osmotic and ionic adjustment, root-system remodeling, and rhizosphere-assisted resource capture [22,23,24]. These modules are introduced here only briefly and discussed mechanistically in later sections to avoid unnecessary repetition. These effects are now supported by a growing body of crop-based evidence. Exogenous GR24 has enhanced antioxidant activity in rice, preserved chlorophyll and photosynthetic rate in apple (*Malus hupehensis* Rehder), maintained gas-exchange traits in wheat, maize, and grapevine (*Vitis vinifera* L.), alleviated salt-induced photodamage and excessive ROS accumulation in cucumber (*Cucumis sativus* L.), and promoted osmotic adjustment, proline accumulation, and heat tolerance in tomato [18,21,22]. In *Arabidopsis thaliana*, for example, GR24 treatment markedly improves survival under drought compared to untreated controls. This illustrates how SL-associated signaling can shift stress outcomes [18,21]. Thus, SLs are now best understood as dual-function molecules. They operate both as external ecological signals in the rhizosphere and as internal hormones that reprogramme plant development, physiology, and stress acclimation across multiple organizational levels [25,26].

Taken together, current evidence indicates that SLs aid abiotic stress adaptation mainly through hormonal crosstalk, rather than a single linear pathway. SLs interact with abscisic acid, auxin, cytokinin, ethylene, and gibberellin, which together define regulatory axes for stomatal behavior, root–shoot allocation, branching, senescence, and stress-responsive growth [27,28,29]. Yet, several key questions remain. In particular, the endogenous, stress-specific roles of SLs must be distinguished from effects obtained after application of synthetic analogs such as GR24. Endogenous evidence currently indicates that SLs regulate drought mainly through stomatal control and ABA-linked water economy, salinity through ion-homeostasis and salt-sensitive mutant phenotypes, heavy-metal stress through redox protection and growth maintenance in SL-deficient lines, and heat and cold through stress-induced activation of SL-biosynthetic and signaling genes. The shared and stress-specific signaling nodes connecting SLs with other hormone pathways are only partly defined. Current evidence points to several candidate nodes, including the D14–MAX2/D3–D53/SMXL signaling module, KAI2/MAX2-related butenolide signaling, PIN1-mediated auxin transport, ABA-linked guard-cell and miR156 signaling, CK-linked lateral-root regulation, ET–auxin control of root-hair development, and GA–ABA balance during thermal responses. However, how these nodes are dynamically regulated under combined or sequential stresses remains poorly resolved; therefore, future studies should integrate transcriptomics, proteomics, metabolomics, hormone profiling, genetic tools, single-cell/nucleus RNA sequencing, and spatial transcriptomics to distinguish conserved SL–hormone crosstalk modules from stress-specific, tissue-specific, and cell-type-specific regulatory nodes. Much current evidence still relies on exogenous GR24 in controlled settings; therefore, throughout this review, we distinguish three levels of evidence: direct endogenous evidence from mutants, gene-silencing, grafting, or native SL quantification; mixed endogenous–exogenous evidence combining pathway activation with GR24 rescue; and analog-only evidence that remains suggestive but not definitive [24,25,26,27]. This review focuses on how SLs interact with these hormones to regulate plant responses to diverse stresses. Particular emphasis is placed on SL biosynthesis and signaling, hormone crosstalk, stress-specific mechanisms, and molecular, physiological, biochemical, and morphological processes underlying stress resilience. By integrating these aspects, this review aims to clarify current knowledge, highlight key mechanistic gaps, and support future research and crop-improvement strategies for unstable climates.

## 2. Review Methodology and Literature Search Strategy

This review was prepared using a structured literature search to ensure broad and balanced coverage of strigolactone biology and abiotic stress responses. Relevant publications were identified from major scientific databases, including Web of Science, Scopus, PubMed, and Google Scholar, using combinations of keywords such as “strigolactone”, “strigolactones”, “GR24”, “abiotic stress”, “drought”, “salinity”, “salt stress”, “heavy metal stress”, “heat stress”, “cold stress”, “hormonal crosstalk”, “abscisic acid”, “auxin”, “cytokinin”, “ethylene”, “gibberellin”, “root architecture”, “flowering”, and “plant growth and development”. Priority was given to peer-reviewed research articles, recent review papers, and mechanistic studies involving SL biosynthesis, signaling mutants, endogenous SL measurements, exogenous SL analogs, hormone interactions, and crop stress responses. Bibliometric trends were qualitatively examined by analyzing publication frequency, recurring keywords, major research themes, and highly cited studies related to SL-mediated stress tolerance. The selected literature was then organized thematically into sections covering SL structure, biosynthesis, signaling, hormonal crosstalk, growth and developmental regulation, stress-specific responses, mechanistic resilience, and agricultural applications.

## 3. Strigolactones: Structural Diversity, Biosynthesis, Transport, and Signaling

To interpret how SLs contribute to plant development and abiotic stress acclimation, their chemistry, biosynthetic origin, spatial distribution, and signal perception must be considered as a single integrated framework. SL activity depends not only on the presence of the hormone, but also on the structural class produced, the site of synthesis, the manner of transport within the plant or exudation into the rhizosphere, and the conversion of the signal into downstream developmental and transcriptional responses [30,31,32,33]. Accordingly, this section first outlines SL structural diversity, then summarizes the conserved and divergent steps of SL biosynthesis, followed by transport and homeostatic regulation, the core perception/signaling module, and finally the stress-responsive regulation of SL-related genes and pathway activity under adverse environments [33,34,35].

### 3.1. Structural Diversity of Strigolactones

Strigolactones are carotenoid-derived butenolide molecules that occur as a chemically diverse family of natural compounds, with more than thirty structures now described across plant species [30,31,34]. They are broadly classified into canonical and non-canonical forms. Canonical SLs possess the characteristic ABCD ring system, in which a tricyclic lactone scaffold (ABC rings) is linked through an enol-ether bridge to the butenolide D-ring; non-canonical SLs lack one or more of the A, B, or C rings, but still retain the enol-ether-linked D-ring that is essential for biological activity [20,21,22,23,24]. Canonical SLs are further separated into strigol-type and orobanchol-type molecules according to the stereochemistry of the B–C ring junction, whereas non-canonical SLs include compounds such as carlactone, carlactonoic acid, methyl carlactonoate, lotuslactone, heliolactone, zealactone, and avenaol [11,22,34]. Structure–activity analyses indicate that the D-ring together with the enol-ether linkage forms the conserved bioactive module, while changes in the surrounding scaffold can markedly alter branching inhibition, parasitic seed germination, and hyphal-branching activity in arbuscular mycorrhizal fungi [35,36,37]. Thus, SL structural diversity should be viewed not as a purely chemical feature, but as a mechanistic basis for functional specificity across species and ecological contexts. Differences between canonical and non-canonical SLs, strigol-type and orobanchol-type stereochemistry, and species-specific derivatives can influence receptor selectivity, rhizosphere signaling, parasitic-weed germination, AMF responsiveness, branching inhibition, and stress-adaptive outputs [31,32].

### 3.2. Biosynthesis of Strigolactones

Despite their structural diversity, the early stages of SL biosynthesis are highly conserved and begin in plastids with all-trans-β-carotene [31,33,38]. The first committed step is catalyzed by DWARF27 (D27), an iron-containing β-carotene isomerase that converts all-trans-β-carotene into 9-cis-β-carotene [31,33]. This intermediate is then cleaved sequentially by CAROTENOID CLEAVAGE DIOXYGENASE 7 (CCD7; MAX3/D17/HTD1/RMS5/DAD3) and CCD8 (MAX4/D10/RMS1/DAD1) to generate carlactone (CL), the central precursor from which both canonical and non-canonical SLs are derived [31,33,37,38]. The importance of this plastidial core is supported by mutant analyses in *Arabidopsis thaliana*, *Oryza sativa*, *Pisum sativum*, and *Petunia hybrida*, in which disruption of D27, CCD7, or CCD8 results in the characteristic high-branching SL-deficient phenotype [31,37]. After CL is formed, the pathway diverges extensively. In *A. thaliana*, MORE AXILLARY GROWTH 1 (MAX1/CYP711A1) oxidizes CL to carlactonoic acid (CLA), which can then be methylated to methyl carlactonoate (MeCLA) and further modified by LATERAL BRANCHING OXIDOREDUCTASE (LBO) into downstream oxidized derivatives [31,35,39,40]. In rice, several MAX1/CYP711A paralogues act sequentially and channel the pathway toward canonical products such as 4-deoxyorobanchol and orobanchol, whereas in species such as tomato, cotton (*Gossypium arboreum* L.), and cowpea (*Vigna unguiculata* L.), CYP722C enzymes contribute to species-specific downstream diversification [31,35]. Accordingly, the route from β-carotene to CL is largely conserved, whereas the steps beyond CL or CLA are lineage-specific and account for the major source of SL structural diversity. This downstream diversification is functionally important because different MAX1/CYP711A and CYP722C-dependent products may generate species-specific SL profiles, allowing plants to tune developmental regulation, microbial recruitment, parasitic-weed risk, and stress responses to their ecological niche [41,42,43]. Recent evidence also suggests that SL homeostasis includes a catabolic layer, because CARBOXYLESTERASE15 (CXE15) and related enzymes can degrade canonical and non-canonical SLs, thereby fine-tuning endogenous hormone abundance [35,44].

### 3.3. Transport and Regulation of SL Homeostasis

SLs are synthesized predominantly in roots and, in some species, in basal stem tissues, from where they can either be exuded into the rhizosphere or transported acropetally to aerial organs [32,45]. Physiological and grafting studies showed that root-derived SL-related signals move through the xylem, linking below-ground biosynthesis with shoot architectural control [37,46]. At the same time, rhizosphere release is an active and spatially regulated process rather than passive leakage. In *Petunia hybrida*, the ATP-binding cassette transporter PLEIOTROPIC DRUG RESISTANCE 1 (PDR1) is required for efficient SL export into the rhizosphere and also contributes to internal movement toward the shoot [32,41]. pdr1 mutants retain near-normal SL content in root tissues but exhibit strongly reduced levels in root exudates, increased shoot branching, and impaired interaction with arbuscular mycorrhizal fungi, indicating that transporter-dependent partitioning is essential for both in planta signaling and rhizosphere communication [41]. At the whole-plant level, SL homeostasis is further controlled by nutrient status and feedback regulation. Phosphate deficiency is the most robust inducer of SL biosynthesis and exudation, but nitrate deficiency and, in some systems, sulfur deficiency can also enhance SL production [33,45,46,47]. These conditions commonly increase the transcription of D27, CCD7, CCD8, and, in some species, MAX1, while elevated expression of biosynthetic genes in SL-deficient and SL-insensitive mutants supports the existence of feedback regulation [33,37,45]. Thus, SL homeostasis is the integrated outcome of biosynthesis, transport, exudation, perception-dependent feedback, and catabolism, allowing plants to match SL output to developmental and environmental demand [33,35,37].

### 3.4. Perception and Signal Transduction

**Figure 1:**
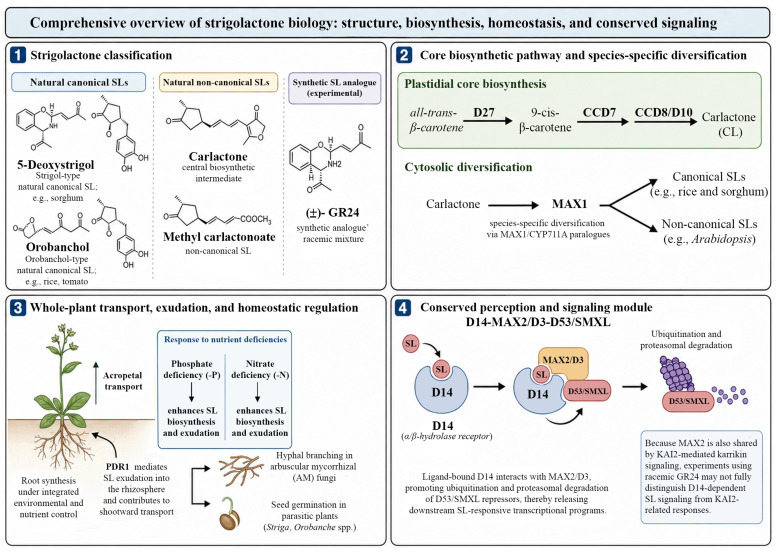
Structural, biosynthetic, transport, and signaling framework of strigolactones. The figure summarizes the major structural classes of strigolactones (SLs), including canonical, non-canonical, and synthetic analog forms, and outlines the conserved biosynthetic route from β-carotene to carlactone followed by species-specific diversification. It also shows SL transport, root exudation, rhizosphere signaling, and the core D14–MAX2/D3–D53/SMXL signaling module. The figure emphasizes that endogenous SL signaling, synthetic analog responses such as GR24, and MAX2-associated KAI2-related signaling should be interpreted separately.

SL perception is centered on DWARF14 (D14), an α/β-hydrolase receptor that binds bioactive SLs and initiates the core signaling cascade [35,42]. Upon ligand perception, D14 undergoes a conformational transition that enables interaction with the F-box protein MORE AXILLARY GROWTH 2 (MAX2), known as DWARF3 (D3) in rice, which functions as the substrate-recognition component of a SKP1–CULLIN–F-box (SCF) E3 ubiquitin ligase complex [35,42,43]. The key output of this pathway is the ubiquitination and proteasomal degradation of the transcriptional repressors DWARF53 (D53) in rice and the homologous SUPPRESSOR OF MAX2 1-LIKE proteins SMXL6, SMXL7, and SMXL8 in A. thaliana [35,42,43]. Once these repressors are removed, downstream SL-responsive programs are released from inhibition, leading to altered shoot branching, root-system remodeling, leaf senescence, and broader transcriptional reprogramming. Mechanistically, the D14–MAX2/SCF–D53/SMXL module therefore constitutes the conserved signaling core through which SL perception is translated into developmental and stress-associated outputs. This module is also a primary molecular entry point for SL–hormone crosstalk, because changes in D14/MAX2-dependent repressor degradation can alter downstream ABA-, auxin-, CK-, ET-, and GA-responsive transcriptional programs under stress [35,42,43]. A critical nuance is that MAX2 also participates in the perception of karrikins and related butenolides through KARRIKIN INSENSITIVE 2 (KAI2). Therefore, D14-dependent SL signaling and KAI2-related signaling should not be treated as interchangeable pathways. D14 primarily mediates canonical SL responses linked to shoot branching, root architecture, endogenous SL perception, and hormone crosstalk, whereas KAI2-related signaling is more strongly associated with karrikin/KL perception, seedling establishment, germination-related responses, environmental adaptation, and stress-associated developmental plasticity. As a result, experiments based on racemic GR24 can blur the distinction between D14-dependent SL signaling and KAI2-related signaling [35,43]. This distinction is especially relevant in stress biology, where both pathways may influence overlapping outputs such as seedling establishment and abiotic stress responsiveness. Under natural conditions, D14-dependent signaling is expected to be most important when plants respond to endogenous SL fluctuations caused by nutrient deficiency, drought, salinity, or temperature stress, whereas KAI2-related signaling may be more relevant to soil- and light-associated environmental cues, smoke-derived or endogenous KL-like compounds, early seedling vigor, and stress priming. Because MAX2 is shared by both pathways, max2 phenotypes cannot be interpreted as exclusively SL-specific without comparison with d14 and kai2 mutants or pathway-selective ligands. A schematic synthesis of SL structural classes, biosynthesis, transport, and core perception/signaling is summarized in Figure 1 [31,35]. The major genes and proteins involved in SL biosynthesis, transport, perception, D14-dependent signaling, and KAI2-related signaling are summarized in Table 1.

### 3.5. Stress-Responsive Regulation of SL Pathways

The SL pathway is itself highly stress-responsive, indicating that SL biology under adverse environments depends not only on downstream hormone action, but also on dynamic regulation of pathway activity at the levels of biosynthesis, transport, and perception [33,35,37]. The best-characterized example is nutrient stress: under phosphate starvation and, to a lesser extent, under nitrate deficiency, plants commonly accumulate higher SL levels and exude more SLs into the rhizosphere [33,45,46]. This response is usually accompanied by enhanced transcription of D27, CCD7, CCD8, and, in some systems, MAX1, demonstrating that pathway activation occurs already at the biosynthetic level [33,45]. Beyond nutrient deprivation, drought, salinity, temperature extremes, and oxidative stress can also alter SL-related genes in a species- and context-dependent manner; importantly, these changes provide endogenous evidence that the SL pathway is stress-responsive, rather than merely responsive to externally supplied GR24 [35,36,37]. In several systems, stress-associated changes have been reported for CCD7, CCD8, MAX1, D14, and MAX2, consistent with the view that environmental signals can prime the SL pathway to support root growth, symbiotic competence, water economy, and redox balance. Importantly, stress may alter not only total SL abundance but also the relative composition of SL types, suggesting that plants may adjust SL structural profiles to match species-specific developmental demands and rhizosphere conditions [35,36]. Additional regulatory layers are provided by interactions with auxin, abscisic acid, sugar signaling, and microRNA-regulated circuits, which together help explain why SL output is tightly coupled to developmental stage and environmental context [33,37,47]. Thus, stress-responsive regulation of SL pathways should be considered an integral component of plant acclimation rather than a secondary consequence of stress exposure.

Overall, the functional specificity of SLs likely emerges from three linked layers: structural class, biosynthetic context, and ecological receiver. At the structural level, canonical and non-canonical SLs, as well as strigol- and orobanchol-type stereoisomers, differ in biological activity. At the biosynthetic level, lineage-specific enzymes, such as MAX1/CYP711A paralogues and CYP722C proteins, shape the SL profile of each species. At the ecological level, the same SL compound may have different consequences depending on whether the receiver is the plant itself, AMF, parasitic weed seeds, rhizobia, or other rhizosphere microbes. Therefore, SL diversity should be interpreted as a chemical language through which plants coordinate endogenous development and external ecological interactions under nutrient limitation and abiotic stress.

## 4. Strigolactones in Plant Growth–Stress Coordination

A central reason why SLs have emerged as key regulators of abiotic stress adaptation is that they do not act solely as stress-associated signals; rather, they coordinate growth, development, and resource economy under adverse environments. Under nutrient deficiency, drought, salinity, or thermal stress, plant acclimation requires the reorganization of plant form, selective restraint of costly growth programs, redirection of assimilates, and improved exploitation of the rhizosphere. Thus, SLs act through three integrated modules—shoot restraint, root-system adjustment, and rhizosphere support—rather than through repeated, separate stress-response pathways [48,49,50]. This integrative role places SLs at the interface between plant development and stress physiology, allowing environmental limitation to be translated into adaptive structural and functional plasticity [48,51].

### 4.1. Root–Shoot Architectural Remodeling Under Stress

One of the best-established functions of SLs is the remodeling of plant architecture in response to environmental constraints. In shoots, SLs suppress axillary bud outgrowth and reduce branching and tillering, thereby limiting the formation of additional sinks when water or nutrients are insufficient to support expansive growth [51,52]. This role was first recognized through the classical high-branching phenotypes of Arabidopsis thaliana, pea (*Pisum sativum* L.), rice, and petunia (*Petunia hybrida* L.), in which defects in SL biosynthesis or signaling result in excessive shoot branching [51,52]. Mechanistically, SL-mediated inhibition of bud growth is linked to transcriptional repression of branch-promoting programs and modulation of PIN-FORMED1 (PIN1)-dependent auxin transport from buds into the main stem [53,54]. By weakening auxin export from lateral buds, SLs reinforce apical dominance and restrain branch proliferation [53].

Under stress, this architectural restraint is adaptive because it lowers demand for carbon, nitrogen, phosphorus, and water, while preserving structural economy [48,50]. In cereals, the same principle applies to tillering, a major determinant of canopy structure and grain production. Under phosphate deficiency in rice, SL signaling suppresses tiller bud outgrowth and contributes to an architecture better suited to low-resource environments [55]. SLs are also implicated in the control of branch and tiller angle through interactions with gravitropic and auxin-related pathways such as LAZY1, LOOSE PLANT ARCHITECTURE1 (LPA1), and IDEAL PLANT ARCHITECTURE1 (IPA1) in rice [56]. Thus, SLs influence both branch number and spatial shoot deployment, traits that affect light interception, hydraulic safety, and resource use under stress.

Below ground, SL effects are more context-dependent than their shoot effects, but several trends are consistent. In many species, SLs stimulate primary root elongation, crown root extension, and root hair development, thereby increasing soil exploration and the absorptive interface for water and nutrients [49,57,58]. Exogenous GR24 and intact SL signaling promote root hair elongation in Arabidopsis, rice, and other systems, and support primary root growth under nutrient limitation [49,57]. Root hairs are especially important under phosphorus deficiency because they enlarge the effective surface area for uptake of immobile nutrients. In contrast, SL effects on lateral roots are conditional: under nutrient-sufficient conditions, SLs often suppress lateral root formation, whereas under phosphate starvation, they can alter lateral-root patterning through cytokinin-linked pathways [57,59]. These architectural outputs are closely tied to auxin transport and sensitivity, and in some cases to ethylene- and cytokinin-associated signaling [49,54,59]. Overall, SL-mediated architecture can be summarized as stress-compatible developmental plasticity: reduced shoot demand combined with context-dependent root foraging [48,49,50].

### 4.2. Resource Allocation and Developmental Plasticity

The architectural functions of SLs are tightly linked to resource allocation. Under favorable conditions, plants can sustain rapid branch formation, leaf expansion, and reproductive development; under stress, however, these programs can become maladaptive if resource demand exceeds supply. SLs appear to shift plants from growth maximization toward stress-compatible allocation, in which carbon, water, and mineral nutrients are directed toward maintenance, uptake, osmotic adjustment, and protective metabolism rather than unnecessary organ proliferation [48,50,60]. By restricting bud outgrowth and limiting additional branches or tillers, SLs reduce the number of developing organs competing for assimilates [51,52]. At the same time, SL-dependent stimulation of root elongation and root hair development enhances access to limiting soil resources [49,57]. Thus, SLs regulate not only where the plant grows, but also where resources are preferentially invested.

Evidence from nutrient-deficiency responses supports this allocation model. Under phosphate and nitrate limitation, increased SL biosynthesis is associated with reduced shoot branching, altered tillering, enhanced mycorrhizal interaction, and modified root development, reflecting coordinated reallocation of biomass and signaling effort toward nutrient acquisition [55,61]. Likewise, under drought or osmotic stress, SLs have been linked to stomatal regulation, improved water economy, and conservative growth strategies, indicating that the same hormonal system can coordinate structural and physiological priorities [48,50]. SL-mediated developmental plasticity also extends to senescence and tissue prioritization, potentially supporting nutrient recycling when organ maintenance becomes costly under stress [48,62]. Thus, this subsection defines SLs as allocation signals, whereas specific antioxidant, osmotic, ionic, and photosynthetic mechanisms are discussed only in the stress-specific sections below [50,60].

### 4.3. Rhizosphere Interactions and Mycorrhizal Support

A distinctive feature of SL biology is that its adaptive role extends beyond the plant body into the rhizosphere. Unlike most classical phytohormones, SLs function both as endogenous regulators and as exuded signals that modify plant–microbe interactions [61,63]. This external signaling role is especially important under nutrient and water limitation, when acclimation depends not only on intrinsic plant traits but also on recruitment of beneficial soil partners. The best-known example is the induction of hyphal branching in arbuscular mycorrhizal fungi (AMF). Root-exuded SLs stimulate pre-symbiotic fungal growth and increase the probability of host-root colonization [61]. Once established, AMF expand the functional absorptive surface of roots and improve acquisition of immobile nutrients, particularly phosphorus, while also contributing indirectly to nitrogen acquisition, hydraulic performance, and stress buffering [61,63]. This explains why SL biosynthesis and exudation commonly increase under phosphate deficiency and, in some species, under nitrogen deficiency [61,64].

This rhizosphere function complements SL-mediated architectural responses. Under nutrient-poor conditions, plants can combine reduced shoot proliferation and enhanced root hair development with microbial recruitment that expands nutrient capture beyond the root surface [61,63]. Because AMF can also improve drought tolerance through enhanced water uptake and better nutritional status, SL-mediated symbiosis may support resilience under combined nutrient and water stress [61,63]. Beyond AMF, SL exudation may influence rhizobia, plant-growth-promoting bacteria, and other non-AMF microbes, but causal evidence remains limited and field-dependent; soil type, nutrient status, moisture, genotype, microbial legacy, and management practices likely determine whether these interactions are beneficial, neutral, or undesirable [60,64]. Therefore, SL-mediated rhizosphere signaling should be presented as a promising but context-dependent component of stress tolerance rather than as a universally established field mechanism.

Taken together, the evidence indicates that SLs help plants confront stress not by a single mechanism, but by harmonizing several complementary layers of adaptation. They suppress excessive shoot proliferation, reinforce absorptive root traits, redirect internal resources toward survival-compatible functions, and strengthen rhizosphere support under limiting environments.

## 5. Crosstalk Between Strigolactones and Phytohormones Under Abiotic Stress

The architectural and ecological functions described in the previous section indicate that the contribution of SLs to abiotic stress adaptation is largely expressed through hormonal networks rather than through a linear, isolated pathway. Through crosstalk with abscisic acid (ABA), auxin, cytokinin (CK), ethylene (ET), and gibberellin (GA), SLs influence stomatal behavior, branching, root plasticity, senescence, rhizosphere support, and developmental timing under adverse environments [11,13]. This network-based view avoids treating SLs as a separate stress hormone and instead positions them as regulators of hormone-guided acclimation [25]. Key molecular entry points include D14-MAX2/D3-D53/SMXL-dependent transcriptional derepression, MAX2/KAI2-related butenolide signaling, PIN1-dependent auxin transport, ABA-linked SnRK2-ABF/AREB and miR156 modules, CK signaling through AHK-AHP-ARR components, ET-auxin regulation through EIN3/EIL-ERF pathways, and GA-ABA control through DELLA-, PIF-, NCED-, and GA-metabolism-related targets [40,41,42,43]. Because MAX2 is shared by SL and KAI2-related pathways, SL-specific interpretation requires support from d14, d53/smxl, SL-biosynthetic mutants, native SL measurements, or stereochemically defined SL ligands, whereas KAI2-related outputs require kai2, smxl2/smxl3/smxl4, or KL/karrikin-responsive evidence. This framework explains why SL effects are context-dependent but repeatedly converge on adaptive outputs, including tighter stomatal control, reduced shoot proliferation, optimized root foraging, altered senescence, and improved compatibility between growth and survival [35,65].

### 5.1. Strigolactone–ABA Crosstalk in Stomatal Regulation and Water-Deficit Signaling

Among the currently known interactions of SLs, crosstalk with ABA is most directly relevant to drought and osmotic stress because both pathways converge on stomatal behavior, leaf water status, and post-stress recovery [11,66]. Although SLs and ABA both originate from carotenoid cleavage products, their stress relevance lies mainly in signaling integration rather than shared metabolic origin [13,65]. Functionally, the strongest evidence concerns guard-cell regulation. In *Arabidopsis thaliana*, SL-deficient or SL-insensitive mutants display impaired stomatal control and enhanced water loss, whereas GR24 treatment or intact SL signaling promotes ABA-associated stomatal closure and improves dehydration tolerance [11,35]. In barley, ABA modulates endogenous SL levels during tillering, suggesting bidirectional communication rather than a strictly downstream SL response [67]. Rice data further show that SL-ABA interaction is stress-stage dependent: SL production increases strongly under mild drought but less under prolonged severe drought, when ABA accumulation becomes dominant [66]. In tomato, a drought-induced SL-miR156 module increases guard-cell ABA sensitivity and delays stomatal reopening after rewatering [67], while grafting evidence indicates that root SL status contributes to systemic drought signaling [68]. Together, these findings support a concise model in which SLs modulate ABA responsiveness, recovery kinetics, and water economy rather than simply amplifying ABA signaling. Mechanistically, this interaction likely involves PYR/PYL receptors, PP2Cs, SnRK2 kinases, ABF/AREB transcription factors, RD29A/RD29B, RAB18, LEA proteins, aquaporins, antioxidant-related genes, and the SL-miR156 module [11,35,66].

### 5.2. Strigolactone–Auxin Crosstalk in Root Architecture and Branching Control

The interaction between SLs and auxin remains the mechanistic cornerstone of SL-regulated architecture. Auxin establishes apical dominance and shapes shoot and root patterning through polar transport, whereas SLs alter the developmental conditions under which auxin flux sustains bud activation or root branching [26,69]. In shoots, SLs weaken PIN1-dependent auxin export from lateral buds, reducing bud competitiveness and reinforcing apical dominance [53,69]. This mechanism helps explain SL-associated branch restraint under phosphate deficiency and other resource-limited conditions, where continued shoot proliferation would increase sink demand [51,55]. Below ground, SL-auxin crosstalk is more context dependent. In A. thaliana, GR24 alters root system architecture and root-hair elongation, while in rice endogenous SL accumulation under nitrate and phosphate deficiency is associated with auxin-regulated remodeling of primary and lateral root development [49,54,55]. Under phosphate stress, SLs may suppress lateral root initiation in some contexts but redirect root growth toward more foraging-efficient patterns in others, indicating that SLs tune auxin outputs rather than simply oppose auxin activity [54,59]. Auxin can also act upstream of SL output, as shown in apple, where auxin-responsive regulation of SL synthesis is linked to mycorrhizal formation under drought [12]. Overall, SL-auxin crosstalk forms a regulatory loop that shapes apical dominance, lateral bud inhibition, and root-system plasticity under stress. At the molecular level, this loop involves PIN1-dependent auxin canalization, TIR1/AFB-Aux/IAA-ARF signaling, BRC1/TB1/FC1-like TCP transcription factors in shoot buds, and auxin-responsive genes controlling primary-root elongation, lateral-root initiation, and root-hair development [12,26,69].

### 5.3. Strigolactone–Cytokinin Crosstalk in Root–Shoot Balance

Cytokinin-SL interaction is especially important for root–shoot balance because the two pathways often impose contrasting developmental priorities. CK generally promotes cell division, bud activation, and shoot growth, whereas SLs favor branch restraint, below-ground investment, and conservative allocation under stress [13,35,70]. This antagonism is clearest in branching control, where CK promotes bud activation while SLs reinforce bud dormancy under nutrient-deficient and drought-compatible developmental conditions [26,71]. However, the relationship is not simple opposition: in *Arabidopsis*, SLs influence lateral root development through the CK signaling network, indicating that CK also participates in SL-mediated root patterning [59,71]. Stress studies support this developmental-priority model. In tall fescue, exogenous 6-benzyladenine suppressed drought-induced FaD14 and FaMAX2 expression, implying antagonism between CK signaling and SL-mediated drought adjustment [12]. In rice, CK and SL also act antagonistically during mesocotyl elongation in darkness [72]. Thus, SL-CK crosstalk can be summarized as a resource-allocation switch that balances shoot activation against root function and stress restraint. Mechanistically, this interaction likely involves AHK receptors, AHP phosphotransfer proteins, and type-A/type-B ARR transcriptional regulators intersecting with SL-controlled bud dormancy, lateral-root patterning, and allocation programs [13,35,71].

### 5.4. Strigolactone–Ethylene Crosstalk in Root Hair Development and Stress Acclimation

The interaction between SLs and ET is most evident in local growth responses, particularly root-hair development and tissue remodeling. Ethylene regulates stress-induced epidermal differentiation, senescence, and growth adjustment, whereas SLs intersect with this network most clearly in root-associated traits [13,54]. In A. thaliana, SLs interact with ET and auxin to control root-hair elongation, and GR24 promotes root-hair growth in a signaling-dependent manner [73]. Because root hairs enlarge the absorptive surface and improve phosphorus and water acquisition, this interaction is relevant to nutrient stress and drought acclimation [54,73]. Although ET can regulate adventitious root initiation independently of SLs in some contexts [74], overlap between the pathways in root hairs, senescence-linked adjustment, and stress-responsive growth suggests convergence on shared morphological outputs. In this model, ET mediates rapid local adjustment, whereas SLs connect local remodeling to whole-plant allocation and stress-compatible architecture. Mechanistically, SL-ET crosstalk likely involves EIN2, EIN3/EIL1, ERF transcription factors, and auxin-dependent RHD6/RSL-type root-hair regulators, linking SL signaling, ET-auxin balance, epidermal differentiation, and absorptive capacity under nutrient or water limitation [13,35,74].

### 5.5. Strigolactone–Gibberellin Crosstalk in Growth Modulation and Temperature Responses

Crosstalk between SLs and GA provides an additional layer of growth control because GA promotes elongation, seed germination, and developmental progression, whereas SLs often favor growth restraint and architectural economy [13,75]. A key mechanistic point is that GA can act upstream of SL output. In rice, GA signaling regulates SL biosynthesis, showing that the two pathways are directly connected rather than merely parallel [76]. This relationship is important under abiotic stress because environmental cues that alter GA status may secondarily reshape endogenous SL levels, affecting tillering, elongation, and resource allocation. The interaction is especially relevant to temperature responses. Under high-temperature germination, SL application can lower the ABA/GA ratio, partly by repressing NCED9 and stimulating GA accumulation, thereby alleviating thermo-inhibition [34,75]. In lupine seedlings, GR24 improved heat tolerance by enhancing antioxidant activity, increasing proline accumulation, and protecting photosynthesis [34]. In perennial species such as *Jatropha curcas* L., GA promotes shoot branching, indicating that SL-GA crosstalk also affects branch control and temperature-linked developmental adjustment. Mechanistically, this interaction may involve GID1-mediated GA perception, DELLA turnover, PIF-associated growth regulation, and ABA/GA metabolic genes such as NCED, GA20ox, GA3ox, and GA2ox, which together influence germination, elongation, thermotolerance, and stress-dependent growth restraint [77].

### 5.6. Integrated Hormonal Model of SL-Mediated Adaptation

**Figure 2:**
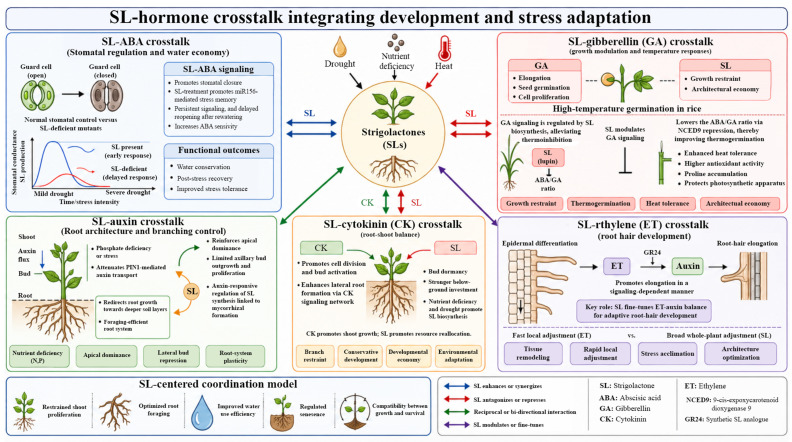
Strigolactone–phytohormone crosstalk under abiotic stress. The figure illustrates how SLs interact with abscisic acid, auxin, cytokinin, ethylene, and gibberellin to coordinate stress acclimation and developmental adjustment. These interactions regulate stomatal behavior, root–shoot architecture, root-hair development, senescence, thermo-responsive growth, and stress-compatible resource allocation. The response curve is schematic and represents conceptual trends rather than quantitative measurements.

Taken together, the evidence indicates that SLs function as hormonal coordinators under abiotic stress rather than as isolated growth regulators. Their interaction with ABA links SLs to stomatal closure, drought signaling, water economy, and recovery; their interaction with auxin governs apical dominance, bud repression, and root plasticity; crosstalk with CK shapes root–shoot balance; interaction with ET contributes to root-hair elongation and local remodeling; and interaction with GA modulates elongation, germination, and temperature-responsive development [11,13,26,35,54]. This integrated model explains why SL-mediated responses are context dependent yet converge on a limited set of adaptive outputs: restrained shoot proliferation, stronger below-ground investment, improved water control, stress-compatible resource allocation, and coordination between development and survival [78]. Identifying which SL-associated transcriptional changes are shared across stresses and which are stress specific will require multi-omics analyses combined with mutant, gene-silencing, grafting, reporter-line, and CRISPR/Cas-based validation. This is particularly important under combined or sequential stresses, where SL crosstalk is likely to be dynamically reweighted: drought–salinity may prioritize ABA-linked stomatal control and ion-transport nodes; nutrient deficiency followed by drought may prioritize SL-auxin-CK control of root–shoot allocation; heat followed by drought may shift signaling toward GA-ABA balance, HSP expression, and antioxidant defense; and cold followed by oxidative stress may favor ABA-CBF, membrane-stability, and redox-related nodes [48,65]. From a translational perspective, manipulation of SL pathways is therefore unlikely to affect a single trait in isolation; rather, it may reshape interdependent physiological and architectural responses. A conceptual summary of this hormone-centered coordination is presented in Figure 2, and the major interaction nodes are synthesized in Table 2.

## 6. Stress-Specific Roles of Strigolactones in Abiotic Stress Mitigation

Abiotic stresses impair plant performance through partially overlapping but physiologically distinct forms of injury. Drought lowers tissue hydration, stomatal conductance, and carbon assimilation; salinity combines osmotic stress with ion toxicity; heavy metals disrupt nutrient balance and intensify oxidative injury; heat destabilizes membranes, proteins, and photosynthetic machinery; and low temperature reduces membrane fluidity, chloroplast efficiency, and osmotic balance. Across these stresses, recurring injury markers include ROS accumulation, membrane damage, chlorophyll loss, photosynthetic decline, and reduced biomass or yield; therefore, the following subsections emphasize stress-specific SL functions rather than repeating all shared protective outputs for each stress [1,3,7,11,28,35]. In crop systems, the quantitative impact can be substantial: low temperature may reduce rice yield by about 30–40%, while drought and heat can reduce grain yield by roughly 9–10% in sensitive systems [79]. To avoid over-interpreting analog-based studies, this section distinguishes endogenous SL functions from exogenous GR24 responses and, where possible, separates D14-dependent SL signaling from MAX2/KAI2-related butenolide signaling. This distinction is important because racemic GR24 can activate both D14- and KAI2-associated pathways, whereas natural stress responses may involve endogenous SLs, KL-like signals, and shared MAX2-dependent signaling. Endogenous evidence is strongest when stress phenotypes are altered in SL-biosynthetic or signaling mutants, native SL levels change under stress, or SL-related genes such as CCD7, CCD8, MAX1, D14, and MAX2 are required for tolerance [35,79,80,81,82,83,84]. Where source studies reported explicit quantitative changes, those values are highlighted below.

### 6.1. Drought Stress

Drought constrains soil water uptake, depresses cell turgor, restricts stomatal aperture, limits CO_2_ assimilation, and accelerates oxidative injury. At the whole-plant level, it commonly reduces shoot elongation, leaf expansion, lateral root development, chlorophyll content, RWC, net photosynthesis, and biomass, while increasing electrolyte leakage, MDA, and H_2_O_2_ [3,11,35]. For example, water deficit suppresses chlorophyll content and photosynthetic rate in grapevine and intensifies membrane injury in winter wheat [80,81]. Successful acclimation therefore depends mainly on coordinated stomatal control, tissue water retention, root-system adjustment, osmotic protection, and antioxidant capacity. Conversely, the SL-biosynthetic mutants max3 and max4, as well as the signaling mutant max2, show accelerated water loss under dehydration compared with wild type, providing endogenous evidence that SLs are required for drought avoidance through stomatal closure, leaf water-loss control, ABA responsiveness, and post-drought recovery rather than through antioxidant protection alone [3,11]. Exogenous SL application or enhanced SL signaling mitigates drought injury across several species. In *Arabidopsis thaliana*, foliar GR24 increased survival under severe drought from 29% in untreated controls to 100% in treated plants [3]. In grapevine, GR24 alleviated drought-induced decline in chlorophyll content by 34% and photosynthetic rate by 28%, and improved plant water status by 22% [80]. In winter wheat, foliar and root GR24 application improved membrane stability by 40% and reduced MDA accumulation by 35% [81]. Another dryland wheat study showed that GR24 increased leaf ABA concentration by 30.6% at 60% field capacity and 28.9% at 50% field capacity, increased root biomass by 36.8% under moderate drought and 54.5% under severe drought, and raised grain yield under moderate drought by 34.9% [82]. In maize, 15 µM GR24 increased root length by 34.3%, shoot fresh weight by 42.7%, chlorophyll content by 127%, and net photosynthetic rate by 34.5%, while reducing H_2_O_2_ by 32.5%, O_2_^−^ by 33.3%, and MDA by 55.2% [1]. In Napier grass, 3 µM GR24 increased net photosynthetic rate by 41%, stomatal conductance by 88%, transpiration by 37%, and restored Fv/Fm from 0.65 to 0.79 [2]. In tomato, combined SA+GR24 under short-term drought increased RWC by 19% and decreased H_2_O_2_ by 35% and TBARS by 52% [5]. Mechanistically, SL-mediated drought resilience integrates ABA-sensitive stomatal regulation, root-based water acquisition, osmotic adjustment, and redox buffering. GR24-treated wheat showed 54.5% greater root biomass under severe drought and accumulated 37.6% more proline and 43.6% more soluble sugars, while maize showed strong induction of antioxidant enzymes, including APX (+101.4%) and CAT (+53.2%) [1,35,48,81,85]. Thus, under drought stress, the most distinctive SL-linked functions are stomatal regulation, ABA sensitivity, root-based water acquisition, and post-drought recovery, whereas antioxidant and osmotic responses are shared protective modules across stresses. Together, these responses combine water-saving and water-acquiring strategies with cellular protection.

### 6.2. Salinity Stress

Salinity impairs growth through an early osmotic phase and a later ionic phase. The osmotic phase restricts water uptake, leaf expansion, and stomatal aperture, whereas the ionic phase causes Na^+^ and Cl^−^ toxicity, disrupts Na^+^/K^+^ balance, destabilizes membranes, accelerates chlorophyll degradation, and suppresses photosynthesis [1,3,7]. In maize, 180 mM NaCl reduced cob length, cob diameter, grains per cob, grain weight per cob, and 100-grain weight, with stronger damage in the salt-sensitive PH4CV genotype than in Zheng58 [3]. In tomato, 150 mM NaCl reduced shoot and root growth by 70% and 30%, respectively, while increasing proline and H_2_O_2_ [1]. SLs mitigate salt injury by improving ion homeostasis, membrane stability, antioxidant activity, and photosynthetic performance. In rice, GR24 enhanced germination, plant height, root length, chlorophyll content, photosynthetic rate, and antioxidant enzyme activity, while reducing MDA accumulation, with 1–1.2 µM being most effective [8,82]. In rapeseed, 0.18 µM GR24 improved shoot and root growth, PSII quantum yield, and antioxidant activity under 100–200 mM NaCl [83]. In ornamental sunflower, 0.01 mg L^−1^ GR24 increased SOD, CAT, and POD activities by 74–176% and reduced shoot Na^+^ by 30–35% under 150 mM NaCl [7]. Dose optimization remains important; in apple, 100 µM GR24 reduced seedling wilting to 13.3%, whereas 10 µM and 1 mM were less effective [85,86]. In cucumber, 5 µM GR24 improved K^+^ retention and reduced Na^+^ accumulation, with H_2_O_2_ and Ca^2+^ signaling acting downstream [7]. At the molecular level, SLs regulate ion transport, ROS detoxification, and osmolyte metabolism. In apple, GR24 upregulated Na^+^ transporter genes (MhCHX15, MhSOS1, MhCAX5), moderated K^+^ transporters (MhNHX1, MhNHX2), enhanced SOD, POD, and CAT activities, and reduced ROS and lipid peroxidation under saline–alkaline stress [85,86]. In tomato, SL-deficient ccd7 mutants show heightened salt sensitivity that is rescued by GR24, confirming that endogenous SL biosynthesis contributes directly to salt tolerance. Unlike drought, where endogenous SLs mainly support stomatal water conservation, salinity-specific SL functions center on Na^+^/K^+^ balance, ion-transporter regulation, ROS detoxification, and preservation of photosynthetic function [1]. In wheat, 10 µM GR24 increased grain yield by 50%, reduced H_2_O_2_ and MDA, and enhanced APX and POX activity under 100 mM NaCl [4]. Transcriptomic evidence further showed upregulation of antioxidant genes (TaAPX, TaGPX), ion transporters (TaSOS1, TaAKT2, TaHAK), and stress-responsive transcription factors, alongside downregulation of TaP5CS [4]. SLs also interact with trehalose metabolism, ABA signaling, and AM symbiosis under salinity [2,5,6]. Collectively, the salinity-specific contribution of SLs centers on ion homeostasis, especially Na^+^/H^+^ antiporters, K^+^ transport systems, and Na^+^/K^+^ balance, while antioxidant protection, photosynthetic maintenance, and osmotic adjustment remain shared stress-protective outputs [1,2,3,4,5,6,7,8,87,88,89].

### 6.3. Heavy-Metal Stress

Heavy metals impose toxicity through direct oxidative injury and secondary nutrient imbalance. Metals such as Cd, Pb, Cr, As, Ni, Cu, and Zn disrupt chlorophyll biosynthesis, stomatal function, nutrient uptake, and membrane stability, leading to chlorosis, growth inhibition, electrolyte leakage, and biomass loss [28,35,79]. In lettuce, 300 ppm Pb reduced leaf and root fresh weight by 56% and 80%, respectively, while increasing H_2_O_2_ and MDA by 78.6% and 90.3% [1]. In melon, 300 µM CdCl_2_ reduced root length, surface area, and tip number by 63%, 38%, and 61%, respectively [5]. In rice, arsenate caused stronger biomass reduction in SL-deficient mutants than in wild type, and Ni toxicity in pepper suppressed growth, disturbed nutrient and hormone balance, and downregulated photosynthesis- and water-transport-related genes [8,48]. SLs mitigate metal toxicity in multiple systems. In lettuce under Pb stress, 20 µM GR24 increased leaf biomass by 156% and root biomass by 464%, restored chlorophyll content by approximately 95%, enhanced N, P, K, and Fe accumulation, and reduced Pb accumulation and oxidative damage by more than 80% [1]. Under Cr stress in tomato, GR24 improved growth and activated the ascorbate–glutathione cycle [4]. In sweet wormwood, GR24 increased biomass and chlorophyll content while reducing Cd accumulation by up to 56% and increasing artemisinin production [3]. In soybean, GR24 decreased Cd accumulation by 35–42% through glyoxalase-mediated detoxification [9]. Similar benefits have been reported in barley, switchgrass, melon, radish, and pepper under different metal stresses [2,5,6,7,10,48]. Genetic evidence also supports a central role for SL signaling, as rice d10 and d17 and barley hvd14.d mutants accumulate more metal, suffer greater oxidative damage, and show stronger growth inhibition than wild type [6,8]. At the mechanistic level, the heavy-metal-specific contribution of SLs is best framed around redox buffering, metal transport or partitioning, nutrient balance, and detoxification metabolism. SLs also promote metabolic reprogramming, including jasmonic acid and flavonoid biosynthesis, preserve chlorophyll and photosystem components, and restore nutrient homeostasis disrupted by metal competition. However, compared with drought and salinity, transporter-specific mechanisms underlying SL-regulated metal homeostasis remain less resolved, particularly for HMA, NRAMP, ZIP, ABC transporter, and aquaporin families under different exposure regimes.

### 6.4. High-Temperature Stress

High temperature destabilizes cellular membranes, denatures proteins, accelerates chlorophyll degradation, disrupts PSII and photosynthetic electron transport, increases REL and lipid peroxidation, and reduces growth and productivity [1,7,28]. Thermotolerance therefore requires antioxidant defense, osmoprotectant accumulation, photosynthetic protection, and induction of heat shock proteins [28,90]. Available evidence indicates that SLs alleviate heat damage, although the dataset remains narrower than for drought or salinity [28]. In tall fescue, 0.01 µM GR24 promoted crown-root and leaf elongation under heat stress, increased cell-cycle-related gene expression, and decreased auxin-transport-related genes such as TIR1, PIN1, PIN2, and PIN5 [91,92]. In tomato, 1, 3, and 9 µM GR24 increased HSP70 accumulation, ABA synthesis, and antioxidant components including SOD, APX, GR, MDAR, and DHAR, while decreasing heat sensitivity, MDA, and H_2_O_2_ [90]. Endogenous SL metabolism is also heat responsive in tomato: solanacol increased by 68.7% after 3 h of heat treatment, and heat induced root transcription of CCD7, CCD8, MAX1, and MAX2 [90]. Silencing CCD7, CCD8, MAX1, or MAX2 increased heat susceptibility, with transcript reductions of 76–80% in silenced lines relative to controls [90]. The induction of CCD7, CCD8, MAX1, and MAX2, together with endogenous solanacol accumulation, indicates that the SL pathway itself is heat responsive; therefore, endogenous SLs likely support thermotolerance through HSP-linked protein protection, antioxidant defense, ABA-associated responses, and maintenance of root and shoot growth [90]. Additional evidence from narrow-leafed lupine shows that 3 µM rac-GR24 improved seed resilience to high temperature, increased SOD, proline, glyoxalase I/II activity, and PIabs, and reduced lipid peroxidation, POD activity, and ABS/RC [93]. In Arabidopsis, GR24 alleviated thermo-inhibition during germination by decreasing the ABA/GA ratio and promoting GA and CK accumulation [94]. Overall, SLs are promising regulators of heat tolerance, but broader crop-level and field validation remains needed [28,90].

### 6.5. Low-Temperature Stress

Low-temperature stress, including chilling and freezing, reduces membrane fluidity, slows enzymatic activity, impairs chloroplast metabolism, and disrupts photosynthetic electron transport [2,25]. These effects increase REL, ROS accumulation, and lipid peroxidation, leading to pigment loss, lower photosynthetic efficiency, inhibited growth, and yield penalties; in rice, low temperature has been associated with yield reductions of approximately 30–40%. Effective cold acclimation depends on membrane stabilization, osmotic adjustment, antioxidant defense, and activation of cold-responsive signaling pathways. Available evidence indicates that SLs contribute positively to cold acclimation, although fewer studies are available than for drought or salinity [60,70]. In tomato, cold treatment induced transcription of CCD7, CCD8, MAX1, and MAX2 and increased endogenous solanacol accumulation, providing direct evidence that cold stress activates the native SL pathway; the cold-specific SL role involves membrane stabilization, antioxidant defense, ABA-linked CBF signaling, and protection of photosynthetic tissues [91]. Exogenous GR24 further enhanced cold tolerance by increasing SOD, APX, GR, MDAR, and DHAR activities, increasing leaf ABA content, and inducing NCED6 and CBF1 expression. These findings link SL-mediated cold protection to both redox regulation and ABA-associated cold signaling [94]. Additional quantitative evidence comes from rape, where 0.1 µmol L^−1^ GR24 increased SOD, POD, CAT, and APX activities, proline, and soluble protein contents, while decreasing H_2_O_2_, MDA, and relative conductivity [91]. GR24 also upregulated MPK3, MPK6, ICE1, and COR, indicating activation of MAPK- and cold-responsive transcriptional networks. In mung bean, 1 and 10 µM GR24 enhanced RWC, soluble sugars, and proline while reducing O_2_^•−^, H_2_O_2_, and MDA under chilling stress. GR24 also preserved PSII performance and physiological status under low temperature [91]. Genetic evidence further supports endogenous SL signaling: pea SL-deficient and SL-response mutants developed more leaves than wild type after dark chilling, whereas *Arabidopsis* max4-1 and max2-1 showed reduced rosette area under dark chilling. Recent *Arabidopsis* work also indicates that the SL receptor pathway promotes freezing tolerance through MAX2-dependent degradation of WRKY41, thereby releasing CBF/DREB1 expression and strengthening cold acclimation [90,91].

Mechanistically, SL-mediated cold tolerance relies on antioxidant reinforcement, osmolyte accumulation, membrane preservation, and activation of regulators such as ICE1, COR, CBF1, and DREB1 [95]. Compared with drought and heat, cold-stress studies emphasize osmolyte accumulation and membrane stability, reflecting the importance of hydration and membrane integrity under chilling and freezing. Table 3 classifies stress-specific SL evidence by stress type, species, genetic material, SL/GR24 application, gene-expression or native-SL profile, physiological outcome, and evidence category. Overall, SLs are promising regulators of cold acclimation, but broader validation across crops and field-relevant cold regimes remains necessary.

## 7. Mechanistic Basis of Strigolactone-Mediated Abiotic Stress Resilience

Across drought, salinity, heavy-metal toxicity, heat, and cold, SL-mediated resilience is best understood as a coordinated network that links stress signaling with physiological differentiation, growth, development, flowering regulation, and reproductive transition, rather than as a single protective pathway. These responses include changes in gene expression, antioxidant defense, osmotic and ionic adjustment, membrane stabilization, photosynthetic protection, and root–rhizosphere adaptation [1,3,11]. This mechanistic view is important because similar visible outcomes, such as improved survival, higher chlorophyll content, or stronger roots, can arise through different pathways depending on stress type, species, developmental stage, and hormone status [35,48]. Overall, SLs appear to improve stress tolerance by synchronizing molecular signaling, biochemical buffering, physiological stability, developmental plasticity, and growth-related differentiation processes, including shoot branching, tillering, root-system formation, leaf senescence, flowering, and reproductive development [7,28].

### 7.1. Regulation of Stress Signaling and Transcriptional Reprogramming

A primary mechanistic layer of SL-mediated resilience is the reprogramming of stress-responsive signaling and gene expression. The canonical SL signaling module, built around DWARF14 (D14), MAX2/D3, and the D53 or SMXL6/7/8 repressors, links ligand perception to ubiquitin-dependent repressor degradation and downstream transcriptional outputs [5,30,42]. Under abiotic stress, this module extends beyond branch inhibition: stress-induced changes in D27, CCD7, CCD8, MAX1, D14, MAX2, D53, and SMXL-related components provide an endogenous basis for SL-dependent transcriptional regulation. Future transcriptomic, proteomic, metabolomic, single-cell/nucleus RNA-sequencing, and spatial-transcriptomic studies should determine whether these genes act as conserved SL-responsive hubs or as stress-, tissue-, and cell-type-specific regulatory nodes [1,11,99]. In tomato, heat and cold induce CCD7, CCD8, MAX1, and MAX2, whereas silencing these genes increases thermal sensitivity, indicating that SL-pathway activation is functionally required for full acclimation rather than merely correlated with stress exposure [91,94]. SL-associated transcriptional outputs can be grouped into six recurring modules: hormone signaling, root development, antioxidant/redox regulation, ion transport, osmotic/metabolic protection, and heat- or cold-responsive transcriptional programs [1,11,35]. These modules include antioxidant genes encoding SOD, CAT, APX, and POD; ion-homeostasis genes such as SOS1, NHX, HKT, and H^+^-ATPase-related transporters; osmotic-protection genes such as P5CS and LEA; heat-response genes such as HSFs and HSP70/HSP90; cold-response genes such as CBF/DREB and COR/RD-type genes; and root-development regulators linked to PIN-mediated auxin transport and root-hair differentiation [100].

These targets likely represent downstream branches of specific SL-hormone nodes: ABA-related pathways regulate stomatal closure and drought recovery; auxin/PIN1 modules regulate bud outgrowth and root architecture; CK-related nodes influence lateral-root patterning and shoot activation; ET-auxin nodes regulate root-hair elongation; and GA-ABA nodes modulate germination, elongation, and temperature-responsive growth. Thus, SL signaling reshapes the transcriptional landscape so that developmental and physiological priorities become compatible with adverse conditions. Under combined or sequential stresses, this regulation is likely temporal and hierarchical: early stress may activate SL biosynthesis and perception genes, whereas later stress phases may redirect signaling toward ABA-responsive guard-cell genes, ion transporters, antioxidant enzymes, HSPs, CBF-related cold-response genes, and root-development regulators [101]. In horticultural and crop systems, GR24 and endogenous SL signaling have been linked to genes associated with antioxidant defense, osmotic adjustment, developmental restraint, ion transport, and temperature acclimation [12,88,94]. Accordingly, SL-hormone crosstalk should be interpreted as a dynamic network whose dominant nodes vary with stress order, duration, intensity, tissue type, developmental stage, and cellular identity. Single-cell/nucleus RNA sequencing and spatial transcriptomics can further resolve whether SL biosynthesis, perception, transport, and hormone-crosstalk modules act preferentially in guard cells, vascular tissues, root epidermal cells, meristems, mesophyll cells, or rhizosphere-associated root cell populations under abiotic stress [99].

### 7.2. Maintenance of Redox Homeostasis and Antioxidant Defense

One of the most consistently supported mechanisms of SL-mediated tolerance is redox regulation, which includes not only antioxidant scavenging but also the balance between ROS, reactive nitrogen species (RNS), and their signaling dynamics [102]. Drought, salinity, heavy metals, heat, and cold promote ROS accumulation, including H_2_O_2_ and superoxide, which drives lipid peroxidation, chlorophyll degradation, protein oxidation, and membrane injury [1,3,7]. Across species, SL treatment commonly increases SOD, POD, CAT, and APX activities while lowering H_2_O_2_ and MDA [11,28]. Recent redox research further indicates that ROS and RNS are compartment-specific signaling molecules that regulate gene expression, stomatal behavior, programmed cell death, antioxidant activity, and stress adaptation through oxidative and nitrosative networks [102].

Quantitative evidence supports this redox-centered interpretation. GR24 improved antioxidant activity and reduced MDA in salt-stressed rice and rapeseed [82,83], enhanced APX, CAT, POD, and membrane stability in drought-stressed wheat [81], alleviated cadmium injury in switchgrass [87], and lowered REL, H_2_O_2_, and MDA during tomato heat and cold responses [90,94]. Mechanistically, SLs help maintain the redox environment required for membrane, protein, and chloroplast function. This regulation should also be considered within ROS/RNS crosstalk, because H_2_O_2_, nitric oxide (NO), peroxynitrite, and related reactive species regulate stress responses through post-translational modifications such as oxidation, S-nitrosation, and nitration [102]. These processes may influence hormone signaling, calcium-dependent signaling, stomatal closure, antioxidant activation, and root development, adding a regulatory layer to SL-associated resilience [102]. Therefore, SLs support tolerance by buffering both oxidative damage and redox/nitrosative signaling networks involved in stress perception and acclimation.

### 7.3. Osmotic Adjustment, Ion Homeostasis, and Membrane Stability

A second mechanistic foundation of SL-mediated resilience is stabilization of water status, ion relations, and membrane integrity. Under drought and low temperature, plants must maintain tissue hydration and osmotic balance; under salinity and some metal stresses, they must also control toxic ion accumulation and preserve the Na^+^/K^+^ ratio [3,7,35]. The literature consistently links SL treatment with higher proline, soluble sugars, and other compatible solutes, together with lower electrolyte leakage and reduced membrane lipid peroxidation [3,11,81,91]. In grapevine and wheat, GR24 improved RWC, membrane stability, and osmoprotective metabolism under drought [80,81]. In *Brassica rapa* and mung bean under low temperature, GR24 increased proline, soluble proteins, and soluble sugars while reducing H_2_O_2_ and MDA, indicating osmotic buffering and membrane preservation [79]. Under salinity, osmotic stabilization is closely coupled with ion homeostasis. In rice, GR24 improved plant height, root length, chlorophyll content, stomatal conductance, and photosynthetic rate, with 1 µM being the most effective concentration tested [82]. In apple seedlings under salinity-alkalinity stress, 100 µM GR24 reduced wilting from 73.3% to 13.3% and upregulated ion-transport and H^+^-ATPase-related genes, including MhCHX15, MhSOS1, MhCAX5, and MhAHA1/3/9 [85,86]. Together, these findings show that SL-mediated tolerance includes protection of the cellular environment in which metabolism occurs, not only activation of upstream signaling or antioxidant defense.

### 7.4. Protection of Photosynthesis and Metabolic Performance

The preservation of photosynthesis is a major downstream consequence of SL action and a direct route through which SLs sustain growth under stress. Abiotic stress commonly reduces chlorophyll content, PSII efficiency, net photosynthetic rate (Pn), stomatal conductance (Gs), and carbon assimilation [1,3,7]. SL treatment repeatedly alleviates these effects by preserving pigments, gas exchange, and photochemical efficiency. For example, GR24 improved photosynthetic rate and chlorophyll levels in drought-stressed grapevine [80], increased chlorophyll content and gas-exchange traits in salt-stressed rice [82], improved PSII quantum yield in rapeseed [83], and restored chlorophyll and photosynthetic performance in cadmium-stressed switchgrass [87]. Mechanistically, SLs protect photosynthesis through interconnected effects on chloroplast redox balance, stomatal function, water economy, chlorophyll stability, and PSII activity [11,66]. This is especially relevant under salinity and heat, where oxidative damage, ion imbalance, and thermal injury rapidly impair chloroplast function [7,83,90]. Because photosynthesis is both a stress target and a prerequisite for recovery, SL-mediated preservation of chloroplast performance strengthens immediate tolerance and post-stress resilience.

### 7.5. Root System Remodeling, Growth and Developmental Plasticity, and Rhizosphere-Assisted Tolerance

SL-mediated stress resilience also depends on structural, developmental, and ecological mechanisms operating below and above ground. In addition to regulating root architecture, root-hair elongation, and AMF-associated rhizosphere interactions, SLs influence shoot branching, tillering, leaf senescence, flowering regulation, and reproductive transition, thereby helping plants balance vegetative growth, organ differentiation, reproductive timing, and resource allocation under stress [103,104]. Several studies show that SLs positively influence primary root elongation, crown root extension, and root-hair development while promoting arbuscular mycorrhizal fungi (AMF) symbiosis [9,26,48,54,61]. Enhanced root growth and root-hair elongation increase direct absorptive capacity, whereas AMF colonization extends nutrient and water uptake beyond the root surface. Under drought and nutrient deficiency, this dual mechanism improves water capture, phosphorus acquisition, and whole-plant performance [11,61].

Nutrient deficiency stimulates SL biosynthesis and exudation, linking internal stress perception with rhizosphere communication [9,61]. Root-exuded SLs stimulate AMF hyphal branching and help initiate symbiosis, allowing plants to recruit microbial support when soil resources are limiting [9,15,61]. Beyond AMF, SLs may also influence rhizosphere-assisted tolerance by shaping microbial community structure, favoring beneficial bacteria such as rhizobia, and indirectly affecting nutrient cycling, root-zone hydration, and stress-protective metabolism; however, direct causal evidence for these non-AMF microbiome effects remains limited, especially under field conditions [105]. Endogenous SL signaling also remodels root architecture to enhance symbiotic effectiveness. Thus, root plasticity and rhizosphere-assisted tolerance should be viewed as a single mechanistic axis. Future work should combine SL-deficient and SL-signaling mutants, native SL quantification, root-exudate profiling, amplicon/metagenomic sequencing, synthetic microbial communities, and field trials under drought, salinity, nutrient limitation, and combined stresses to test whether SL-shaped microbiomes measurably improve stress tolerance and yield stability. In practical terms, this root-rhizosphere mechanism explains why SLs are strongly induced under nutrient deficiency and why their benefits are prominent in drought- and phosphorus-related stress contexts.

### 7.6. Integrated Mechanistic Framework

**Figure 3:**
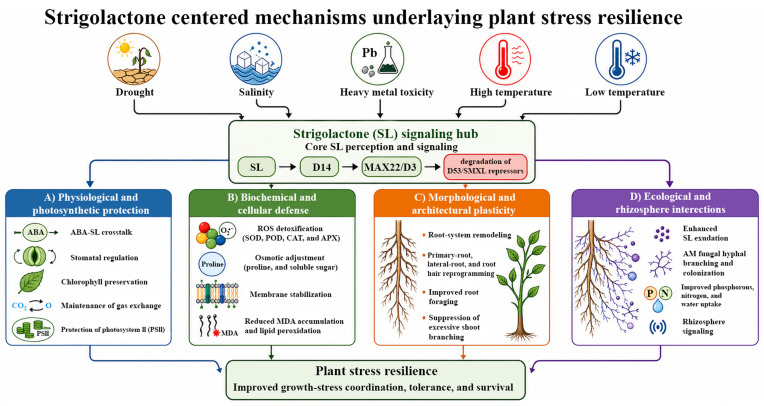
Mechanistic modules of SL-associated abiotic stress resilience. The figure summarizes how SL signaling contributes to plant adaptation under drought, salinity, heavy metal toxicity, heat, and cold. SL-mediated resilience is organized into interconnected modules, including signaling and transcriptional reprogramming, redox and osmotic regulation, ion homeostasis, photosynthetic protection, developmental plasticity, and rhizosphere-assisted resource acquisition. These mechanisms collectively show that SLs enhance stress tolerance through systems-level integration rather than a single isolated pathway.

Taken together, the evidence supports an integrated model in which SL-mediated abiotic stress tolerance results from several mutually reinforcing processes rather than from a single dominant pathway. At the molecular level, D14, MAX2, D53/SMXL6/7/8, D27, CCD7, CCD8, and MAX1 reshape stress-responsive transcriptional programs [1,5,42]. At the biochemical level, SLs reinforce SOD, POD, CAT, APX, and related antioxidant systems while reducing H_2_O_2_ and MDA [7,11,28,96]. At the cellular and physiological levels, they improve osmotic balance, ion homeostasis, membrane stability, chlorophyll retention, PSII efficiency, gas exchange, and metabolic activity [1,3,35,79,80]. At the morphological and ecological levels, they remodel root architecture and strengthen AMF-mediated acquisition of water and nutrients [9,48,61,97]. The mechanistic value of SLs therefore lies in their ability to synchronize signaling reprogramming, antioxidant defense, osmotic and ionic adjustment, photosynthetic protection, developmental plasticity, and root–rhizosphere adaptation into a coherent adaptive state. This systems-level interpretation should be the central message of Figure 3; SLs enhance tolerance not by acting at one site, but by integrating molecular, biochemical, physiological, developmental, and ecological layers of plant acclimation.

## 8. Translational and Agricultural Prospects of Strigolactones

The translational importance of SL biology lies in its role at the intersection of crop architecture, nutrient acquisition, abiotic stress resilience, rhizosphere communication, microbiome recruitment, and parasitic-weed ecology. This combination gives SL research unusual agricultural relevance: the same pathway that restrains excessive branching and improves stress acclimation can also be manipulated to alter root exudation, mycorrhizal recruitment, and host–parasite interactions [8,29,95,106]. Yet this promise comes with an equally distinctive challenge. Because SLs act both inside the plant and in the rhizosphere, agricultural translation requires careful attention to dose, formulation, delivery route, crop genotype, developmental stage, and production environment. Thus, translational work on SLs must be judged not simply by whether a treatment improves stress tolerance in controlled conditions, but by whether it can be converted into robust, scalable, and agronomically safe tools for crop improvement.

### 8.1. Exogenous Application of SL Analogs for Stress Priming

The most immediate translational route for SL biology is the exogenous use of synthetic analogs and mimics, most notably GR24 and related compounds, as stress-priming agents; however, these applications should not be treated as direct substitutes for endogenous SL function unless supported by mutant, rescue, gene-expression, or native-hormone evidence. Controlled-environment studies summarized in earlier sections show that exogenous SL application can improve drought tolerance, protect photosynthesis under salinity and heat stress, strengthen antioxidant defense, and enhance osmotic adjustment across diverse crops [3,7,11,79,80,81,82,83,84,85,86,87,88,89,90,91,92,93,94]. From a translational perspective, the attraction of this approach is clear: unlike breeding or gene editing, exogenous application can be deployed rapidly and does not require permanent genomic modification. It also offers temporal flexibility, as SL analogs can, in principle, be applied at stress-sensitive stages such as germination, early vegetative growth, flowering, or stress recovery. However, the practical value of exogenous SLs depends on moving beyond proof-of-concept physiology to formulation science and field performance. Recent work on agricultural agonists and antagonists emphasizes that the challenge is not merely to synthesize active compounds, but to develop analogs with appropriate stability, soil behavior, receptor selectivity, and compatibility with large-scale delivery [95]. This issue becomes particularly important in the rhizosphere, where rapid degradation, adsorption to soil particles, microbiome-dependent transformation, or non-target biological effects can severely limit efficacy. Field deployment must therefore evaluate not only plant physiological responses but also changes in AMF colonization, bacterial and fungal community composition, nutrient cycling, pathogen pressure, and parasitic-weed stimulation. A notable recent advance is the field-oriented formulation of methyl phenlactonoate (MP3) and Nijmegen-1 for Striga control in Kenya. In laboratory assays, these formulations induced up to 56% germination of Striga seeds, reduced Striga emergence by up to 77% in greenhouse pots, and achieved reductions of up to 80% in mini-field and 65% in field trials, demonstrating that SL-inspired chemistry can be translated into operational agronomy when formulation and delivery are optimized [101]. For abiotic stress management, the same translational logic applies. Analog choice must balance biological potency with cost, shelf life, scalability, and species-specific efficacy. The agronomic future of exogenous SL priming will therefore depend on the development of stable formulations, rational dose windows, stress-stage-specific treatment schedules, and better separation of desired in-plant signaling from undesirable stimulation of parasitic weeds [8,29,95,101].

### 8.2. Genetic Manipulation of SL Biosynthesis and Signaling Pathways

A second translational route is the genetic manipulation of SL biosynthesis, transport, perception, or signaling. This includes classical mutants, transgenic overexpression or suppression lines, transcriptomic profiling, and increasingly, CRISPR/Cas-based editing of pathway genes. Future genetic studies should explicitly compare SL-biosynthetic mutants, d14 mutants, kai2 mutants, max2 mutants, and smxl/d53-related lines under the same stress conditions to separate D14-dependent SL responses from KAI2-related and shared MAX2-dependent responses. These tools should be combined with comparative omics approaches to identify shared SL–hormone crosstalk nodes, such as ABA-linked stomatal regulation and auxin-linked architectural remodeling, as well as stress-specific nodes controlling ion transport, heat-shock responses, cold acclimation, and heavy-metal detoxification. The strongest evidence comes from manipulations of core biosynthetic components such as CCD7, CCD8, MAX1, and transporter genes governing SL exudation. Because these genes influence branching, tillering, root architecture, and rhizosphere signaling, they represent powerful points of intervention for crop design [99,106]. Targeted editing has already demonstrated practical value. In rice, CRISPR/Cas9-mediated disruption of CCD7 generated high-tillering, reduced-height plants and provided proof of principle that precise modification of the SL pathway can re-engineer plant architecture in elite germplasm [99]. In tomato, CRISPR/Cas9-mediated mutagenesis of MAX1 conferred resistance to *Phelipanche aegyptiaca*, demonstrating that editing SL biosynthesis can reduce root-exuded germination cues for parasitic weeds [105]. More recently, genome editing of the tomato SL transporter SlABCG45 conferred broad-spectrum broomrape resistance without an obvious yield penalty in parasite-free fields, and produced an approximately 30% yield increase under Phelipanche-infested field conditions, highlighting how transporter manipulation can decouple resistance and productivity more effectively than broader pathway disruption [100]. In sorghum, editing of CCD7, CCD8, MAX1, and genes in the lgs1 region has likewise been used to reduce host cues for Striga, indicating that SL-centered genome editing can be adapted to cereal systems of direct food-security relevance [102]. Gene manipulation is not limited to loss-of-function strategies. Overexpression studies also illustrate translational potential. In *Arabidopsis*, overexpression of the maize receptor gene ZmD14 enhanced drought resistance and reduced ROS accumulation, suggesting that strengthening SL perception could strengthen stress resilience in crop backgrounds where receptor activity is limiting [103]. The next translational step is to integrate such gene-level interventions with expression profiling, promoter design, and tissue-specific editing so that beneficial traits can be enhanced without incurring excessive penalties in tillering, plant height, or mycorrhizal signaling [103,106].

### 8.3. Strigolactones in Breeding Climate-Resilient Crops

Beyond direct chemical or gene-editing interventions, SL biology offers a framework for breeding climate-resilient crops. The appeal of SL-linked breeding is that the pathway influences several agronomic traits already central to crop improvement: branching and tillering, root system configuration, nutrient-use efficiency, drought adaptation, and parasitic-weed resistance [60,106]. In cereals, the SL pathway is especially relevant to tiller economy and nitrogen responsiveness, while in horticultural crops it shapes root–shoot balance and exudation behavior. This makes SL-linked traits particularly attractive for breeding programs targeting low-input agriculture, drought-prone systems, and fields with chronic parasite pressure. The most promising breeding targets are likely to be those that alter quantitative SL output or tissue-specific SL transport rather than abolishing the pathway entirely. Complete disruption of SL biosynthesis often causes excessive branching and undesirable architectural phenotypes, whereas partial modulation may allow breeders to fine-tune canopy structure, root investment, and exudation without sacrificing adaptation [99,106]. Transporter-associated loci may be especially useful because they can modify rhizosphere signaling with weaker pleiotropic effects on in-plant development. The tomato SlABCG45 case is therefore important not only as a gene-editing proof-of-concept but also as a breeding model for identifying naturally useful alleles that affect exudation rather than total synthesis [100]. In practical breeding terms, SL-linked traits can be integrated into resilience programs through marker-assisted selection, genomic selection, and gene-informed phenotyping. Candidate traits include lower branch density under nutrient limitation, improved root hair and crown-root performance, enhanced mycorrhizal responsiveness, and reduced release of parasite-germination cues [29,60,106]. Because these phenotypes are strongly environment-dependent, breeding for SL-associated resilience will likely require multi-environment testing rather than simple single-site screening. Even so, the pathway provides an unusually coherent set of candidate targets for crops intended for drought-prone, nutrient-poor, or parasite-infested agroecosystems.

### 8.4. Opportunities and Limitations in Agricultural Translation

The opportunity presented by SL research is substantial: one pathway offers leverage over plant architecture, nutrient foraging, abiotic stress acclimation, and root–soil communication. Yet this breadth is also the main source of translational difficulty. Agricultural use of SL analogs or SL-targeted breeding cannot assume uniform benefit because the pathway is strongly dependent on species, developmental stage, nutrient background, and stress regime [8,29,95,106]. A dose that improves drought acclimation in one crop may alter tillering or exudation unfavorably in another, and an analog that is physiologically active in controlled conditions may be unstable or poorly distributed in field soils. A second limitation is chemical instability and formulation sensitivity. Natural SLs are chemically labile, and many synthetic analogs still face constraints related to hydrolysis, photostability, soil persistence, and manufacturing cost [95,101]. The positive field data for formulated MP3 and Nijmegen-1 show that these problems are not insurmountable, but they also demonstrate that translational success depends on formulation rather than just biological activity [101]. A third challenge is biological specificity. Because SLs stimulate mycorrhizal fungi but can also stimulate germination of Striga, Orobanche, and Phelipanche, agricultural deployment must carefully balance beneficial rhizosphere signaling against the risk of worsening parasitic-weed pressure [14,15,29,100]. Genetic approaches have their own limitations. Editing of core biosynthetic genes may improve parasite resistance but can also cause excessive branching, reduced height, or altered reproductive allocation [99,105,106]. Even transporter-based strategies, which are often more elegant, require extensive agronomic validation to ensure that reduced exudation does not compromise mycorrhizal benefits under nutrient-poor conditions [100]. Thus, the main translational lesson is that SL research offers high potential but not a universally transferable solution. The future lies in crop-specific, environment-specific, and trait-specific deployment rather than one-size-fits-all application.

### 8.5. Future Priorities and Research Gaps

Several priorities should define the next phase of translational research on SLs. First, field-scale validation remains disproportionately limited relative to the volume of controlled-environment evidence. Much of the current literature is based on pot experiments, hydroponic systems, or short-term greenhouse assays, whereas agricultural deployment requires multi-location, multi-season, and genotype-aware evaluation under realistic soil, climate, and management conditions [8,29,101,106]. Second, species-specific resolution is essential. The agronomic consequences of SL manipulation are unlikely to be uniform across cereals, legumes, horticultural crops, and woody perennials, because these systems differ substantially in architecture, endogenous SL profiles, receptor sensitivity, root exudation patterns, and rhizosphere interactions. Third, quantitative characterization of endogenous and exuded SLs remains a major bottleneck. Progress in translation will depend on accurate hormone profiling across tissues, developmental stages, and stress regimes, together with clearer experimental separation of D14-mediated SL signaling from KAI2-related butenolide signaling [30,35,106]. Fourth, deeper functional dissection of the D14–MAX2-centered signaling axis, transporter systems, catabolic regulation, and downstream repressors is needed if SL-based pathway engineering is to become predictive rather than largely empirical. Finally, future research should move beyond the broad question of whether SLs are beneficial in principle and instead determine when, where, and in which crop systems SL-based interventions confer a measurable agronomic advantage. The translational future of SLs will therefore depend on integrative research pipelines that combine chemical biology, quantitative physiology, hormone analytics, genetics, genome editing, and field agronomy.

## 9. Conclusions and Future Perspectives

SLs should now be regarded not simply as branching inhibitors or rhizosphere-derived signals, but as integrative regulators of abiotic stress adaptation that connect hormonal crosstalk, developmental plasticity, and multilevel stress defense. Across drought, salinity, heavy metal toxicity, heat, and cold, the evidence synthesized in this review shows that SLs coordinate stomatal regulation, root–shoot balance, antioxidant defense, osmotic and ionic adjustment, membrane stabilization, photosynthetic protection, and rhizosphere-assisted resource acquisition. Their interactions with abscisic acid, auxin, cytokinin, ethylene, and gibberellin further explain why SL-mediated responses are highly context-dependent yet repeatedly converge on a common adaptive outcome: improved compatibility among growth restraint, survival, and recovery under adverse environments. In this sense, SL-mediated stress resilience is best understood as a systems-level phenomenon rather than the consequence of a single linear pathway. At the same time, the translational potential of SL biology remains only partly realized. Future studies should therefore prioritize field validation, crop-specific optimization, and the development of SL-based strategies that improve stress tolerance without compromising yield, disrupting beneficial microbiomes, or increasing the risk of parasitic weeds. In addition, integrated omics and genetic approaches are needed to map SL–hormone crosstalk at higher resolution. Comparative transcriptomics, proteomics, metabolomics, hormone profiling, chromatin-level analyses, single-cell/nucleus RNA sequencing, and spatial transcriptomics should be combined with SL-deficient, SL-insensitive, and hormone-signaling mutants, grafting systems, reporter lines, and CRISPR/Cas-edited materials. These approaches will allow SL-mediated stress responses to be mapped at cell-type and spatial resolution, helping to distinguish organ-wide stress acclimation from localized regulatory events in guard cells, vascular tissues, root tips, epidermal cells, and reproductive tissues. Such studies will help distinguish core SL–hormone modules shared across multiple stresses from stress-specific nodes operating under drought, salinity, heavy-metal toxicity, heat, or cold. Greater integration of physiology, chemical biology, genetics, genome editing, microbiome science, and agronomy will be essential to determine when, where, and in which crop systems SL-based interventions provide a reliable advantage. In particular, paired plant-performance and rhizosphere-microbiome analyses under field conditions are needed to quantify how much of SL-mediated stress tolerance is plant-intrinsic and how much is mediated indirectly through AMF, rhizobia, plant-growth-promoting bacteria, fungal communities, or broader soil-microbial networks. If these priorities are addressed, SL research is well-positioned to contribute meaningfully to the development of climate-resilient, resource-efficient agriculture.

## Figures and Tables

**Table 1:** Core components of strigolactone biosynthesis, transport, perception, D14-dependent signaling, and KAI2-related signaling in representative plant species.

Gene/Protein	Species	Functional Role	Pathway Step	Stress Relevance	Evidence and Interpretive Note	Ref.
*D27*/DWARF27	*Arabidopsis*; rice	β-carotene isomerase converting all-trans-β-carotene to 9-cis-β-carotene	Early plastidial biosynthesis	Induced by nutrient limitation and linked with stress-responsive SL production	Endogenous-pathway evidence: expression and mutant phenotypes support its role upstream of SL-dependent growth and stress responses.	[31,33,37]
*CCD7*/*MAX3*/*D17*/*HTD1*/*RMS5*/*DAD3*	*Arabidopsis*; rice; pea; petunia	Cleaves 9-cis-β-carotene toward SL precursor formation	Early plastidial biosynthesis	Responsive to phosphate starvation and feedback regulation in SL-deficient backgrounds	Strong biosynthetic marker: changes indicate pathway activation, but stress tolerance must be confirmed with mutants, rescue, or native SL measurements.	[33,37,45]
*CCD8*/*MAX4*/*D10*/*RMS1*/*DAD1*	*Arabidopsis*; rice; pea; petunia	Converts the *CCD7* product to carlactone	Early plastidial biosynthesis	Central node in feedback-controlled SL biosynthesis under nutrient and stress signals	Endogenous SL-deficiency evidence: *ccd8*/*max4*/*d10*-type mutants are useful for testing causal stress functions beyond GR24 responses.	[31,33,37]
*MAX1*/*CYP711A1* and *CYP711A* paralogues	*Arabidopsis*; rice	Oxidize carlactone/CLA and direct production of canonical and non-canonical SLs	Downstream biosynthesis and diversification	Shapes species-specific SL profiles relevant to branching, nutrient foraging, AMF signaling, and stress adaptation	Key structure–function node: downstream products differ among species; therefore, SL abundance alone may not predict biological output.	[31,33,39]
*CYP722C*	Tomato; cotton; cowpea	Converts CLA-derived intermediates into species-specific canonical SLs	Downstream diversification	May influence crop-specific rhizosphere signaling, parasitic-weed risk, and stress-associated SL profiles	Species-specific evidence needed: functional consequences under abiotic stress remain less resolved than core *D27*/*CCD7*/*CCD8* steps.	[31,35]
*CLAMT* and *LBO*	*Arabidopsis*	*CLAMT* methylates CLA to *MeCLA*; *LBO* converts *MeCLA* to downstream oxidized derivatives	Late non-canonical SL metabolism	Fine-tunes non-canonical SL pools associated with branching and developmental plasticity	Added interpretive caution: late-pathway products should not be treated as functionally equivalent to canonical SLs or GR24.	[35,40]
*CXE15*/CARBOXYLESTERASE15	*Arabidopsis*	Degrades canonical and non-canonical SLs	Catabolism/homeostasis	Adds a degradation layer to development- and stress-linked SL homeostasis	Homeostatic control: catabolism may explain transient or tissue-specific SL responses under stress, but direct stress studies remain limited.	[35,44]
*PDR1*/ABCG transporter	Petunia	Mediates SL exudation into the rhizosphere and contributes to shootward movement	Transport/exudation	Regulates partitioning between internal hormonal signaling and external rhizosphere communication	Ecological specificity: transporter-mediated exudation can promote AMF interaction but may also increase parasitic-weed germination risk.	[37,41]
*D14*	*Arabidopsis*; rice; petunia	α/β-hydrolase receptor for canonical SL perception	Ligand perception	Connects endogenous SL fluctuations with shoot branching, root architecture, hormone crosstalk, and stress-associated outputs	D14-dependent evidence is required before assigning a stress phenotype to canonical SL signaling.	[35,42]
*MAX2*/*D3*	*Arabidopsis*; rice	F-box component of the SCF ubiquitin ligase complex	Signal transduction shared by *D14* and *KAI2* pathways	Required for degradation of repressors and full SL/*KAI2*-related responsiveness	Not SL-specific alone: *max2*/*d3* phenotypes require comparison with *d14* and *kai2* mutants or ligand-specific assays.	[35,42,43]
*D53* and *SMXL6*/*SMXL7*/*SMXL8*	Rice; *Arabidopsis*	Transcriptional repressors degraded after SL perception	Repressor removal/transcriptional derepression	Release SL-responsive developmental and stress-associated transcriptional programs	Mechanistic output node: degradation links SL perception to downstream targets involved in branching, roots, senescence, and stress adaptation.	[35,42,43]
*KAI2* and *SMAX1*/*SMXL2*/*SMXL3*/*SMXL4*	*Arabidopsis* and other plants	*KAI2* perceives karrikins/*KL*-like butenolides; *SMAX1*/*SMXL2*/*3*/*4* act as downstream repressors	*KAI2*-related signaling module	Associated with germination, seedling establishment, environmental cue responses, and stress-associated developmental plasticity	Newly emphasized: *KAI2*-related signaling must be separated from *D14*-dependent SL signaling, especially in rac-GR24 or *max2*-based studies.	[35,43]
GR24/rac-GR24 and stereochemically defined SL analogs	Experimental tool across many species	Synthetic ligands used to probe SL-related responses	Exogenous analog evidence	Often improves stress traits such as antioxidant activity, root growth, stomatal behavior, and photosynthesis	Analog-only evidence is suggestive, not definitive: rac-GR24 may activate both *D14*- and *KAI2*-related routes; stereospecific ligands and genetic controls are preferred.	[24,25,26,27,35,43]

Note; *D14*-dependent signaling should be interpreted as canonical SL perception, whereas *KAI2*-related signaling refers to karrikin/KL-responsive pathways. Because *MAX2*/*D3* is shared by both modules, *max2*/*d3* phenotypes and rac-GR24 responses should not be interpreted as exclusively SL-specific without *d14*/*kai2* comparison, *d53*/*smxl* or *smax1*/*smxl* controls, ligand-specific assays, or native-hormone evidence.

**Table 2:** Molecular nodes and evidence strength for strigolactone crosstalk with major phytohormones under abiotic stress.

Hormone Partner	SL-Linked Molecular Node	Downstream Targets/Physiological Processes	Stress Context	Representative Outcome	Evidence Strength and Interpretive Caution	Ref.
Abscisic acid (ABA)	D14–MAX2/D3–D53/SMXL module; ABA-sensitive guard-cell signaling; miR156-linked recovery module; PYR/PYL–PP2C–SnRK2–ABF/AREB targets	Stomatal closure, water-loss control, drought recovery, osmotic adjustment, antioxidant activation, aquaporin and LEA/RD-type responses	Drought, osmotic stress, salinity, rewatering/recovery	Mild drought in rice is associated with a strong increase in SL production, whereas prolonged severe drought shows weaker SL induction with stronger ABA accumulation; tomato SL–miR156 signaling delays stomatal reopening after rewatering.	Relatively strong endogenous/mixed evidence: supported by mutants, grafting/native SL measurements, ABA-response assays, and GR24 rescue. Cell-type priority: guard cells and vascular/root tissues should be resolved using reporter, single-cell, or spatial methods.	[11,35,66,67,68,78]
Auxin	PIN1-mediated auxin canalization; TIR1/AFB–Aux/IAA–ARF signaling; BRC1/TB1/FC1-like TCP factors; auxin-regulated SL biosynthesis	Apical dominance, axillary bud inhibition, tillering, primary/lateral root development, root hair elongation, AMF-associated root adaptation	Nutrient deficiency, drought, salinity-associated architectural adjustment, developmental stress	SLs restrict bud auxin export and reinforce apical dominance; under nutrient stress, SL–auxin interaction redirects root architecture and root-hair development rather than uniformly increasing or suppressing root branching.	Strong developmental mechanism; stress translation is context-dependent. Interpret root outcomes by nutrient status, species, developmental stage, and endogenous-vs.-GR24 evidence.	[12,26,54,55,69]
Cytokinin (CK)	Antagonistic bud activation network; AHK–AHP–ARR phosphorelay; CK-linked lateral-root patterning; 6-BA suppression of *FaD14*/*FaMAX2* under drought	Root–shoot balance, lateral-root patterning, shoot activation/restraint, developmental prioritization under resource limitation	Drought, phosphate deficiency, nutrient limitation, early developmental stress	CK generally favors shoot activation and cell division, whereas SLs favor conservative allocation and branch restraint; in tall fescue, 6-BA suppresses drought-induced *FaD14* and *FaMAX2* expression.	Moderate evidence: clear developmental antagonism, but fewer stress-specific causal tests. Future work should test CK-response reporters and SL mutants under defined stress stages.	[12,13,35,59,71,72]
Ethylene (ET)	EIN2–EIN3/EIL–ERF signaling; ET–auxin root-hair module; RHD6/RSL-type root-hair regulators; overlap with senescence and local tissue remodeling	Root-hair elongation, epidermal differentiation, absorptive surface formation, local growth adjustment, senescence-associated remodeling	Nutrient stress, drought acclimation, salinity-associated root adjustment	GR24 promotes root-hair elongation in an ET- and auxin-associated context, supporting absorptive capacity under stress-prone conditions.	Focused but narrower evidence: strongest for root-hair/epidermal responses; broader whole-plant SL–ET stress claims require endogenous and cell-type-resolved validation.	[13,54,73,74]
Gibberellin (GA)	GA regulation of SL biosynthesis; GID1–DELLA–PIF module; ABA/GA balance; *NCED*, *GA20ox*, *GA3ox*, and *GA2ox*-related targets	Seed germination, elongation, thermoresponsive growth, flowering/reproductive transition, branching and developmental timing	Heat stress, thermo-inhibition, germination, developmental timing, reproductive stress sensitivity	SL treatment can reduce the ABA/GA ratio under high-temperature germination conditions and improve heat-adjusted growth responses; GA can also act upstream of SL biosynthesis in rice.	Mixed evidence: mechanistically plausible and supported in selected systems, but many heat/GA claims remain analog-based and need D14/KAI2-discriminating tests.	[13,34,75,76,77]
Integrated SL–hormone network	D14/MAX2/KAI2 discrimination; tissue-specific hormone modules; dynamic reweighting of ABA, auxin, CK, ET, and GA nodes	Growth–stress coordination, redox and osmotic protection, root–shoot allocation, stomatal control, flowering/reproductive stability, rhizosphere interaction	Combined or sequential drought, salinity, heat, cold, heavy-metal, and nutrient stresses	Dominant nodes likely shift with stress order, intensity, duration, tissue type, developmental stage, and cell identity.	New synthesis: future studies should combine mutants, native SL profiling, hormone reporters, CRISPR/Cas materials, single-cell/nucleus RNA-seq, and spatial transcriptomics to distinguish conserved from stress-specific nodes.	[24,25,26,27,28,29,35,65]

Note: Evidence strength is classified qualitatively to prevent equal weighting of endogenous mutant/native-hormone evidence and analog-only GR24 evidence. Where rac-GR24, *max2*/*d3*, or expression-only data are used, D14-dependent SL signaling should be interpreted cautiously unless supported by D14/KAI2-discriminating genetic or ligand-specific validation.

**Table 3:** Classified evidence for strigolactone-mediated abiotic stress responses based on stress type, species, mutant/genetic material, SL/GR24 application, gene-expression profile, physiological outcome, and evidence category.

Stress	Species	Mutant/Genetic Material	SL/GR24 Application	Gene-Expression or Native-SL Profile	Major Physiological/Biochemical Outcome	Evidence Category	Ref.
Drought	*Arabidopsis thaliana*	SL-biosynthetic mutants *max3* and *max4*; signaling mutant *max2*	Foliar GR24	Mutants show impaired endogenous SL biosynthesis/signaling; ABA-linked stomatal regulation implicated	GR24 increased survival from 29% to 100%; max mutants showed accelerated water loss	Mixed endogenous-exogenous evidence	[3,11]
Drought	Grapevine	Not reported	Exogenous GR24	Not reported	Improved chlorophyll content, photosynthetic rate, and plant water status	Analog-based physiological evidence	[80]
Drought	Winter wheat	Not reported	Foliar/root GR24	ABA accumulation and drought-responsive physiological changes reported	Improved membrane stability, antioxidant defense, root biomass, root length density, and grain yield	Analog-based with hormone-response evidence	[81,92]
Drought	Maize	Not reported	15 µM GR24	Not reported	Increased root growth, chlorophyll, photosynthesis, and antioxidant activity; reduced H_2_O_2_, O_2_^•−^, and MDA	Analog-based physiological/biochemical evidence	[1]
Salinity	Tomato	SL-deficient *ccd7* mutant	GR24 rescue	ccd7 salt-sensitive phenotype rescued by GR24, supporting endogenous SL involvement	Improved salt tolerance and reduced stress injury	Mixed endogenous-exogenous evidence	[1]
Salinity	Rice	Not reported	1–1.2 µM GR24	Not reported	Improved germination, plant height, root length, chlorophyll, photosynthesis, and antioxidant enzymes; reduced MDA	Analog-based physiological/biochemical evidence	[8,82]
Salinity	Rapeseed	Not reported	0.18 µM GR24	Not reported	Improved shoot/root growth, PSII quantum yield, and antioxidant activity; decreased lipid peroxidation	Analog-based physiological evidence	[83]
Salinity	Apple	Not reported	100 µM GR24	Upregulation of *MhCHX15*, *MhSOS1*, *MhCAX5* and H^+^-ATPase-related genes	Reduced wilting; improved ion homeostasis, antioxidant defense, and membrane stability	Analog-based with gene-expression evidence	[85,86]
Salinity	Wheat	Not reported	10 µM GR24	Upregulation of *TaAPX*, *TaGPX*, *TaSOS1*, *TaAKT2*, *TaHAK* and stress-responsive TFs; downregulation of *TaP5CS*	Increased grain yield; reduced H_2_O_2_ and MDA; enhanced APX and POX activity	Analog-based with transcriptomic/gene-expression evidence	[4]
Heavy metal	Rice	SL-deficient mutants *d10* and *d17*	Compared with WT	Mutant phenotype indicates endogenous SL contribution to arsenate tolerance	SL mutants showed greater biomass reduction and stress sensitivity than WT	Endogenous genetic evidence	[8]
Heavy metal	Barley	SL-response mutant *hvd14.d*	Compared with WT	D14-related signaling implicated in metal-stress tolerance	Greater metal accumulation, oxidative injury, and growth inhibition than WT	Endogenous signaling-mutant evidence	[6]
Heavy metal	Lettuce	Not reported	20 µM GR24	Not reported	Increased leaf/root biomass, chlorophyll, and nutrient accumulation; reduced Pb accumulation and oxidative damage	Analog-based physiological/biochemical evidence	[1]
Heavy metal	Tomato	Not reported	GR24	Activation of ascorbate–glutathione cycle-related detoxification responses	Improved growth and reduced oxidative damage under Cr stress	Analog-based biochemical evidence	[4]
Heavy metal	Soybean	Not reported	GR24	Glyoxalase-mediated detoxification activated	Reduced Cd accumulation and improved growth	Analog-based detoxification evidence	[9]
Heavy metal	Pepper	Not reported	20 µM SL treatment	Stress-responsive gene expression modulated under Ni stress	Improved growth, antioxidant capacity, sucrose, and nutrient status	Analog-based with gene-expression evidence	[48]
Heat	Tomato	Silenced *CCD7*, *CCD8*, *MAX1*, and *MAX2* lines	1, 3, and 9 µM GR24	Heat induced *CCD7*, *CCD8*, *MAX1*, *MAX2* and increased endogenous solanacol	Increased HSP70, ABA synthesis, and antioxidant activity; reduced MDA and H_2_O_2_; silencing increased heat sensitivity	Strong endogenous + exogenous evidence	[90]
Heat	Tall fescue	Not reported	0.01 µM GR24	Altered expression of cell-cycle and auxin-transport genes, including TIR1, PIN1, PIN2, and PIN5	Improved crown-root and leaf elongation under heat	Analog-based with gene-expression evidence	[96,97]
Heat	Narrow-leafed lupine	Not reported	3 µM rac-GR24	Not reported	Increased SOD, proline, glyoxalase activity, and photosynthetic performance; decreased lipid peroxidation	Analog-based physiological/biochemical evidence	[89]
Heat	*Arabidopsis thaliana*	Not reported	0.1 and 20 µM GR24	ABA/GA ratio altered through NCED repression and GA/CK accumulation	Alleviated thermo-inhibition during seed germination	Analog-based hormone-response evidence	[98]
Low temperature	Tomato	Not reported	GR24; cold treatment	Cold induced *CCD7*, *CCD8*, *MAX1*, *MAX2* and increased endogenous solanacol; *NCED6* and *CBF1* induced by GR24	Improved antioxidant activity, ABA-associated cold signaling, and photosynthetic protection	Endogenous + exogenous gene-expression evidence	[91,94]
Low temperature	*Brassica rapa*	Not reported	0.1 µmol L^−1^ GR24	*MPK3*, *MPK6*, *ICE1*, and *COR* upregulated	Increased SOD, POD, CAT, APX, proline, and soluble proteins; decreased H_2_O_2_, MDA, and relative conductivity	Analog-based with gene-expression evidence	[79]
Low temperature	Mung bean	Not reported	1 and 10 µM GR24	Not reported	Increased RWC, soluble sugars, proline, and PSII performance; reduced O_2_^•−^, H_2_O_2_, and MDA	Analog-based physiological/biochemical evidence	[79]
Low temperature	Pea and *Arabidopsis thaliana*	SL-deficient and SL-response mutants; max4-1 and *max2-1* mutants	Compared with WT	Mutant phenotypes indicate endogenous SL contribution to chilling tolerance	Altered leaf development and reduced rosette area under chilling	Endogenous mutant evidence	[91,94]

## Data Availability

The original contributions presented in this study are included in the article. Further inquiries can be directed to the corresponding author.

## Abbreviations

ABA, abscisic acid; ABC, ATP-binding cassette; ABCG, ATP-binding cassette subfamily G; ABF/AREB, ABA-responsive element-binding factor/ABA-responsive element-binding protein; ABS/RC, absorption flux per reaction center; AHK, Arabidopsis histidine kinase; AHP, Arabidopsis histidine phosphotransfer protein; AI, artificial intelligence; AMF, arbuscular mycorrhizal fungi; APX, ascorbate peroxidase; ARF, auxin response factor; ARR, Arabidopsis response regulator; As, arsenic; BRC1, BRANCHED1; CAT, catalase; CBF, C-repeat binding factor; CCD, carotenoid cleavage dioxygenase; Cd, cadmium; CK, cytokinin; CL, carlactone; CLA, carlactonoic acid; CLAMT, carlactonoic acid methyltransferase; CO_2_, carbon dioxide; COR, cold-regulated; Cr, chromium; CRISPR/Cas, clustered regularly interspaced short palindromic repeats/CRISPR-associated system; Cu, copper; CXE15, CARBOXYLESTERASE15; CYP, cytochrome P450; D3, DWARF3; D10, DWARF10; D14, DWARF14; D17, DWARF17; D27, DWARF27; D53, DWARF53; DAD, DECREASED APICAL DOMINANCE; DELLA, DELLA-domain growth repressor; DREB, dehydration-responsive element-binding protein; ET, ethylene; Fe, iron; FC1, FINE CULM1; GA, gibberellin; GID1, GIBBERELLIN INSENSITIVE DWARF1; GR24, synthetic strigolactone analog; Gs, stomatal conductance; H_2_O_2_, hydrogen peroxide; HKT, high-affinity potassium transporter; HMA, heavy metal ATPase; HSP, heat shock protein; HSP70/HSP90, heat shock protein 70/90; HTD1, HIGH-TILLERING DWARF1; IAA, indole-3-acetic acid; IPA1, IDEAL PLANT ARCHITECTURE1; KAI2, KARRIKIN INSENSITIVE 2; KL, KAI2 ligand/KAI2-related ligand; LBO, LATERAL BRANCHING OXIDOREDUCTASE; LEA, late embryogenesis abundant; LPA1, LOOSE PLANT ARCHITECTURE1; MAPK, mitogen-activated protein kinase; MAX, MORE AXILLARY GROWTH; MDA, malondialdehyde; MeCLA, methyl carlactonoate; miR156, microRNA156; MPK, mitogen-activated protein kinase; NCED, 9-cis-epoxycarotenoid dioxygenase; NHX, Na^+^/H^+^ exchanger; Ni, nickel; NO, nitric oxide; P5CS, Δ^1^-pyrroline-5-carboxylate synthetase; Pb, lead; PDR1, PLEIOTROPIC DRUG RESISTANCE 1; PIF, phytochrome-interacting factor; PIN, PIN-FORMED; POD, peroxidase; PP2C, type 2C protein phosphatase; PSII, photosystem II; PTM, post-translational modification; PYR/PYL, PYRABACTIN RESISTANCE/PYRABACTIN RESISTANCE-LIKE; RAB18, RESPONSIVE TO ABA 18; RD, responsive to desiccation; REL, relative electrolyte leakage; RHD6, ROOT HAIR DEFECTIVE 6; RMS, RAMOSUS; RNA, ribonucleic acid; RNS, reactive nitrogen species; ROS, reactive oxygen species; RSL, RHD6-LIKE; RWC, relative water content; SA, salicylic acid; SCF, SKP1–Cullin–F-box; sc/snRNA-seq, single-cell/nucleus RNA sequencing; SLs, strigolactones; SMAX1, SUPPRESSOR OF MAX2 1; SMXL, SUPPRESSOR OF MAX2 1-LIKE; SnRK2, SNF1-related protein kinase 2; SOD, superoxide dismutase; SOS1, SALT OVERLY SENSITIVE 1; ST, spatial transcriptomics; TBARS, thiobarbituric acid reactive substances; TCP, TEOSINTE BRANCHED1/CYCLOIDEA/PCF transcription factor; TF, transcription factor; TIR1/AFB, TRANSPORT INHIBITOR RESPONSE1/AUXIN SIGNALING F-BOX; WT, wild type; Zn, zinc; ZIP, ZRT/IRT-like protein.

## References

[1] Dong J., Fu H., Wang Z., Zhang L., Liu Z., Hu Y., Shen F., Wang W. (2025). Mechanisms of Strigolactone-Regulated Abiotic Stress Responses in Plants. Plants.

[2] Khan M.K., Pandey A., Hamurcu M., Vyhnánek T., Zargar S.M., Kahraman A., Topal A., Gezgin S. (2024). Exploring strigolactones for inducing abiotic stress tolerance in plants. Czech J. Genet. Plant Breed..

[3] Qi J., Mao Y., Cui J., Lu X., Xu J., Liu Y., Zhong H., Yu W., Li C. (2024). The role of strigolactones in resistance to environmental stress in plants. Physiol. Plant..

[4] Raza A., Javed R., Zahid Z., Sharif R., Hafeez M.B., Ghouri M.Z., Nawaz M.U., Siddiqui M.H., Husen A. (2021). Strigolactones for Sustainable Plant Growth and Production Under Adverse Environmental Conditions. Plant Performance Under Environmental Stress.

[5] Hossain A., Raza A., Maitra S., Asaduzzaman M., Islam M.R., Hossain M.J., El Sabagh A., Garai S., Mondal M., Abdel Latef A.A.H., Aftab T., Hakeem K.R. (2021). Strigolactones: A Novel Carotenoid-Derived Phytohormone—Biosynthesis, Transporters, Signalling, and Mechanisms in Abiotic Stress. Plant Growth Regulators.

[6] Pandey A., Sharma M., Pandey G.K. (2016). Emerging Roles of Strigolactones in Plant Responses to Stress and Development. Front. Plant Sci..

[7] Khalid M.F., Shafqat W., Khan R.I., Jawaid M.Z., Hussain S., Saqib M., Rizwan M., Ahmed T. (2024). Unveiling the resilience mechanism: Strigolactones as master regulators of plant responses to abiotic stresses. Plant Stress.

[8] Chesterfield R.J., Vickers C.E., Beveridge C.A. (2020). Translation of strigolactones from plant hormone to agriculture: Achievements, future perspectives, and challenges. Trends Plant Sci..

[9] Soliman S., Wang Y., Han Z., Pervaiz T., El-kereamy A. (2021). Strigolactones in Plants and Their Interaction with the Ecological Microbiome in Response to Abiotic Stress. Plants.

[10] Selwal N., Wani A.K., Akhtar N., Kaur M., Jassal P.S. (2023). Molecular insights of strigolactone biosynthesis, signalling pathways, regulatory roles, and hormonal crosstalks in plant systems. S. Afr. J. Bot..

[11] Trasoletti M., Visentin I., Campo E., Schubert A., Cardinale F. (2022). Strigolactones as a hormonal hub for the acclimation and priming to environmental stress in plants. Plant Cell Environ..

[12] Fathi A., Ghadirnezhad Shiade S.R., Shohani F., Asgharipour M.R., Mitra D., Mansouri S., Alymanesh M.R., Taylor N.L., Fazeli A. (2025). Harnessing strigolactones and phytohormone interactions to enhance plant adaptation under climate change. Discov. Plants.

[13] Ameen F., Hussain I., Afzal S., Rasheed R., Ashraf M.A., Iqbal M. (2025). Crosstalk between strigolactones and major hormones in plants under abiotic stresses. S. Afr. J. Bot..

[14] Cook C.E., Whichard L.P., Turner B., Wall M.E., Egley G.H. (1966). Germination of Witchweed (*Striga lutea* Lour.): Isolation and Properties of a Potent Stimulant. Science.

[15] Akiyama K., Matsuzaki K.I., Hayashi H. (2005). Plant sesquiterpenes induce hyphal branching in arbuscular mycorrhizal fungi. Nature.

[16] Gomez-Roldan V., Fermas S., Brewer P.B., Puech-Pagès V., Dun E.A., Pillot J.P., Letisse F., Matusova R., Danoun S., Portais J.C. (2008). Strigolactone inhibition of shoot branching. Nature.

[17] Umehara M., Hanada A., Yoshida S., Akiyama K., Arite T., Takeda-Kamiya N., Magome H., Kamiya Y., Shirasu K., Yoneyama K. (2008). Inhibition of shoot branching by new terpenoid plant hormones. Nature.

[18] Yoneyama K., Brewer P.B. (2021). Strigolactones, how are they synthesized to regulate plant growth and development?. Curr. Opin. Plant Biol..

[19] Shahzad K., Danish S., Mubeen S., Dawar K., Fahad S., Hasnain Z., Ansari M.J., Almoallim H.S. (2024). Minimization of heavy metal toxicity in radish (*Raphanus sativus*) by strigolactone and biochar. Sci. Rep..

[20] Guan Y., Li L., Wang D., Zhou J., Qi W., Cheng Y., Jiang Y., Du Q., Zhang D., Quan M. (2025). Multifaceted functions of strigolactones in annual and perennial plants: Developmental regulation, phytohormone crosstalk and abiotic stresses. Plant Stress.

[21] Sánchez Martín-Fontecha E., Cardinale F., Bürger M., Prandi C., Cubas P. (2024). Novel mechanisms of strigolactone-induced DWARF14 degradation in *Arabidopsis thaliana*. J. Exp. Bot..

[22] Lee J., Seo M.G., Lim Y., Hong S., An J.T., Jeong H.Y., Lee C., Park S.J., Song G., Kwon C.T. (2025). Modulating the strigolactone pathway to optimize tomato shoot branching for vertical farming. J. Integr. Plant Biol..

[23] Karniel U., Koch A., Bar Nun N., Zamir D., Hirschberg J. (2024). Tomato Mutants Reveal Root and Shoot Strigolactone Involvement in Branching and Broomrape Resistance. Plants.

[24] El-Beltagi H.S., Gad M., Khedr N., Abdel-Haleem M., Al Saikhan M.S., Shalaby T.A., El-Mogy M.M., Khedr E.H. (2026). Strigolactones as Integrative Regulators of Plant Adaptation and Resilience to Abiotic Stress. Plant Cell Environ..

[25] Mansoor S., Mir M.A., Karunathilake E., Rasool A., Ştefănescu D.M., Chung Y.S., Sun H. (2023). Strigolactones as promising biomolecule for oxidative stress management: A comprehensive review. Plant Physiol. Biochem..

[26] Sun H., Li W., Burritt D.J., Tian H., Zhang H., Liang X., Miao Y., Mostofa M.G., Tran L.P. (2022). Strigolactones interact with other phytohormones to modulate plant root growth and development. Crop J..

[27] Mehmood I., Wani K.I., Aftab T. (2025). Strigolactone Interplay with Other Phytohormones Under Stressed and Normal Conditions. Strigolactones in Plants.

[28] Alvi A.F., Sehar Z., Fatma M., Masood A., Khan N.A. (2022). Strigolactone: An emerging growth regulator for developing resilience in plants. Plants.

[29] Mehmood I., Wani K.I., Aftab T. (2025). Nature, Structural Diversity, Biosynthetic Pathway, and Strigolactone Transport in Plants. Strigolactones in Plants.

[30] Al-Babili S., Bouwmeester H.J. (2015). Strigolactones, a Novel Carotenoid-Derived Plant Hormone. Annu. Rev. Plant Biol..

[31] Ou H., Xie D., Yao R., Shan X. (2026). Strigolactones: Biosynthesis, transport, perception, and signal transduction. Mol. Plant.

[32] Rehman N.U., Li X., Zeng P., Guo S., Jan S., Liu Y., Huang Y., Xie Q. (2021). Harmony but Not Uniformity: Role of Strigolactone in Plants. Biomolecules.

[33] Zwanenburg B., Pospíšil T. (2013). Structure and Activity of Strigolactones: New Plant Hormones with a Rich Future. Mol. Plant.

[34] Wang Y., Bouwmeester H.J. (2018). Structural diversity in the strigolactones. J. Exp. Bot..

[35] Visentin I., Ferigolo L.F., Russo G., Korwin Krukowski P., Capezzali C., Tarkowská D., Gresta F., Deva E., Nogueira F.T., Schubert A. (2024). Strigolactones promote flowering by inducing the miR319-LA-SFT module in tomato. Proc. Natl. Acad. Sci. USA.

[36] Sağlam S., Özel H., Çevik B., Gerçek Y.C. (2025). Effects of GR24 and 24-eBL on the growth of wheat (*Triticum aestivum* L.) seedlings grown under salt stress. N. Z. J. Crop Hortic. Sci..

[37] Wang Y., Gouaille L., Meng J., Nicolas M., Ogé L., Jiang Z., Crespel L., Ding Y., Le Gourrierec J., Li G. (2026). Decoding the sugar–strigolactone crosstalk: New frontier in plant growth and stress resilience. Hortic. Res..

[38] Saeed W., Naseem S., Ali Z. (2017). Strigolactones Biosynthesis and Their Role in Abiotic Stress Resilience in Plants: A Critical Review. Front. Plant Sci..

[39] Abe S., Sado A., Tanaka K., Kisugi T., Asami K., Ota S., Kim H.I., Yoneyama K., Xie X., Ohnishi T. (2014). Carlactone is converted to carlactonoic acid by MAX1 in *Arabidopsis* and its methyl ester can directly interact with AtD14 in vitro. Proc. Natl. Acad. Sci. USA.

[40] Brewer P.B., Yoneyama K., Filardo F., Meyers E., Scaffidi A., Frickey T., Akiyama K., Seto Y., Dun E.A., Cremer J.E. (2016). LATERAL BRANCHING OXIDOREDUCTASE acts in the final stages of strigolactone biosynthesis in *Arabidopsis*. Proc. Natl. Acad. Sci. USA.

[41] Kretzschmar T., Kohlen W., Sasse J., Borghi L., Schlegel M., Bachelier J.B., Reinhardt D., Bours R., Bouwmeester H.J., Martinoia E. (2012). A petunia ABC protein controls strigolactone-dependent symbiotic signalling and branching. Nature.

[42] Zhou F., Lin Q., Zhu L., Ren Y., Zhou K., Shabek N., Wu F., Mao H., Dong W., Gan L. (2013). D14–SCF^D3^-dependent degradation of D53 regulates strigolactone signaling. Nature.

[43] Wang L., Wang B., Jiang L., Liu X., Li X., Lu Z., Meng X., Wang Y., Smith S.M., Li J. (2015). Strigolactone Signaling in *Arabidopsis* Regulates Shoot Development by Targeting D53-Like SMXL Repressor Proteins for Ubiquitination and Degradation. Plant Cell.

[44] Palayam M., Yan L., Nagalakshmi U., Gilio A.K., Cornu D., Boyer F.D., Dinesh-Kumar S.P., Shabek N. (2024). Structural insights into strigolactone catabolism by carboxylesterases reveal a conserved conformational regulation. Nat. Commun..

[45] Mayzlish-Gati E., De-Cuyper C., Goormachtig S., Beeckman T., Vuylsteke M., Brewer P.B., Beveridge C.A., Yermiyahu U., Kaplan Y., Enzer Y. (2012). Strigolactones are Involved in Root Response to Low Phosphate Conditions in *Arabidopsis*. Plant Physiol..

[46] Kohlen W., Charnikhova T., Liu Q., Bours R., Domagalska M.A., Beguerie S., Verstappen F., Leyser O., Bouwmeester H., Ruyter-Spira C. (2011). Strigolactones are Transported through the Xylem and Play a Key Role in Shoot Architectural Response to Phosphate Deficiency in Nonarbuscular Mycorrhizal Host *Arabidopsis*. Plant Physiol..

[47] Liu W., Kohlen W., Lillo A., Ivanov S., Hartog M., Limpens E., Jamil M., Smaczniak C., Kaufmann K., Yang W.C. (2011). Strigolactone Biosynthesis in *Medicago truncatula* and Rice Requires the Symbiotic GRAS-Type Transcription Factors NSP1 and NSP2. Plant Cell.

[48] Yildirim E., Yuce M., Yaprak E., Ucar S., Aydin M., Turan M., Ghosh T.K., Dolaş Z., Akca M., Oztemiz F. (2026). Strigolactone mitigates nickel toxicity by regulating nutrient uptake, antioxidant defense, vitamins and phytohormones biosynthesis in pepper seedlings. J. Hazard. Mater..

[49] Ruyter-Spira C., Kohlen W., Charnikhova T., Van Zeijl A., Van Bezouwen L., De Ruijter N., Cardoso C., Lopez-Raez J.A., Matusova R., Bours R. (2011). Physiological Effects of the Synthetic Strigolactone Analog GR24 on Root System Architecture in *Arabidopsis*: Another Belowground Role for Strigolactones?. Plant Physiol..

[50] Dawood M.F.A., Eissa M.A., Mir M.I., Muhammad M., Abideen Z., Thabet S.G., Abdel Latef A.A.H. (2026). Strigolactones for boosting plant stress tolerance. Using Stimulants to Improve Plant Health.

[51] Wang J.Y., Chen G.T.E., Alashoor K.F., Al-Babili S., Mukhtar S. (2026). Characterization and Quantification of Strigolactones in Root Exudates. Plant Hormones.

[52] Beveridge C.A., Kyozuka J. (2010). New genes in the strigolactone-related shoot branching pathway. Curr. Opin. Plant Biol..

[53] Crawford S., Shinohara N., Sieberer T., Williamson L., George G., Hepworth J., Müller D., Domagalska M.A., Leyser O. (2010). Strigolactones enhance competition between shoot branches by dampening auxin transport. Development.

[54] Yao T., Xu X., Xie R., Zhou C., Li D. (2026). The biosynthesis and signaling regulation of strigolactones in plants. Plant Sci..

[55] Umehara M., Hanada A., Magome H., Takeda-Kamiya N., Yamaguchi S. (2010). Contribution of Strigolactones to the Inhibition of Tiller Bud Outgrowth under Phosphate Deficiency in Rice. Plant Cell Physiol..

[56] Sang D., Chen D., Liu G., Liang Y., Huang L., Meng X., Chu J., Sun X., Dong G., Yuan Y. (2014). Strigolactones regulate rice tiller angle by attenuating shoot gravitropism through inhibiting auxin biosynthesis. Proc. Natl. Acad. Sci. USA.

[57] Arite T., Kameoka H., Kyozuka J. (2012). Strigolactone Positively Controls Crown Root Elongation in Rice. J. Plant Growth Regul..

[58] Kapulnik Y., Delaux P.M., Resnick N., Mayzlish-Gati E., Wininger S., Bhattacharya C., Sejalon-Delmas N., Combier J.P., Bécard G., Belausov E. (2011). Strigolactones affect lateral root formation and root-hair elongation in *Arabidopsis*. Planta.

[59] Jiang L., Matthys C., Marquez-Garcia B., De Cuyper C., Smet L., De Keyser A., Boyer F.D., Beeckman T., Depuydt S., Goormachtig S. (2016). Strigolactones spatially influence lateral root development through the cytokinin signaling network. J. Exp. Bot..

[60] Barbier F., Fichtner F., Beveridge C. (2023). The strigolactone pathway plays a crucial role in integrating metabolic and nutritional signals in plants. Nat. Plants.

[61] Danish S., Hareem M., Dawar K., Naz T., Iqbal M.M., Ansari M.J., Salmen S.H., Datta R. (2024). The role of strigolactone in alleviating salinity stress in chili pepper. BMC Plant Biol..

[62] Yamada Y., Furusawa S., Nagasaka S., Shimomura K., Yamaguchi S., Umehara M. (2014). Strigolactone signaling regulates rice leaf senescence in response to a phosphate deficiency. Planta.

[63] Li X., Wu G., Lie Z., Aguila L.C.R., Khan M.S., Luo H., Liu X., Liu J. (2025). Microbial community variation in rhizosphere and non-rhizosphere soils of *Castanopsis hystrix* plantations across stand ages. J. For. Res..

[64] Yoneyama K., Xie X., Kusumoto D., Sekimoto H., Sugimoto Y., Takeuchi Y., Yoneyama K. (2007). Nitrogen deficiency as well as phosphorus deficiency in sorghum promotes the production and exudation of 5-deoxystrigol, the host recognition signal for arbuscular mycorrhizal fungi and root parasites. Planta.

[65] Wu F., Gao Y., Yang W., Sui N., Zhu J. (2022). Biological Functions of Strigolactones and Their Crosstalk with Other Phytohormones. Front. Plant Sci..

[66] Haider I., Andreo-Jimenez B., Bruno M., Bimbo A., Floková K., Abuauf H., Ntui V.O., Guo X., Charnikhova T., Al-Babili S. (2018). The interaction of strigolactones with abscisic acid during the drought response in rice. J. Exp. Bot..

[67] Visentin I., Pagliarani C., Deva E., Caracci A., Turečková V., Novák O., Lovisolo C., Schubert A., Cardinale F. (2020). A novel strigolactone-miR156 module controls stomatal behaviour during drought recovery. Plant Cell Environ..

[68] Visentin I., Vitali M., Ferrero M., Zhang Y., Ruyter-Spira C., Novák O., Strnad M., Lovisolo C., Schubert A., Cardinale F. (2016). Low levels of strigolactones in roots as a component of the systemic signal of drought stress in tomato. New Phytol..

[69] Hayward A., Stirnberg P., Beveridge C., Leyser O. (2009). Interactions between Auxin and Strigolactone in Shoot Branching Control. Plant Physiol..

[70] Ferguson B.J., Beveridge C.A. (2009). Roles for auxin, cytokinin, and strigolactone in regulating shoot branching. Plant Physiol..

[71] Marzec M., Melzer M. (2018). Regulation of Root Development and Architecture by Strigolactones under Optimal and Nutrient Deficiency Conditions. Int. J. Mol. Sci..

[72] Hu Z., Yamauchi T., Yang J., Jikumaru Y., Tsuchida-Mayama T., Ichikawa H., Takamure I., Nagamura Y., Tsutsumi N., Yamaguchi S. (2014). Strigolactone and Cytokinin Act Antagonistically in Regulating Rice Mesocotyl Elongation in Darkness. Plant Cell Physiol..

[73] Kapulnik Y., Resnick N., Mayzlish-Gati E., Kaplan Y., Wininger S., Hershenhorn J., Koltai H. (2011). Strigolactones interact with ethylene and auxin in regulating root-hair elongation in *Arabidopsis*. J. Exp. Bot..

[74] Rasmussen A., Hu Y., Depaepe T., Vandenbussche F., Boyer F.D., Van Der Straeten D., Geelen D. (2017). Ethylene Controls Adventitious Root Initiation Sites in *Arabidopsis* Hypocotyls Independently of Strigolactones. J. Plant Growth Regul..

[75] Marzec M. (2017). Strigolactones and Gibberellins: A New Couple in the Phytohormone World?. Trends Plant Sci..

[76] Ito S., Yamagami D., Umehara M., Hanada A., Yoshida S., Sasaki Y., Yajima S., Kyozuka J., Ueguchi-Tanaka M., Matsuoka M. (2017). Regulation of strigolactone biosynthesis by gibberellin signaling. Plant Physiol..

[77] Ni J., Gao C., Chen M.S., Pan B.Z., Ye K., Xu Z.F. (2015). Gibberellin Promotes Shoot Branching in the Perennial Woody Plant *Jatropha curcas*. Plant Cell Physiol..

[78] Wang H., Chen W., Eggert K., Charnikhova T., Bouwmeester H., Schweizer P., Hajirezaei M.R., Seiler C., Sreenivasulu N., Von Wirén N. (2018). Abscisic acid influences tillering by modulation of strigolactones in barley. J. Exp. Bot..

[79] Ma Q., Xia Z., Cai Z., Li L., Cheng Y., Liu J., Nian H. (2019). GmWRKY16 Enhances Drought and Salt Tolerance Through an ABA-Mediated Pathway in *Arabidopsis thaliana*. Front. Plant Sci..

[80] Min Z., Li R., Chen L., Zhang Y., Li Z., Liu M., Ju Y., Fang Y. (2019). Alleviation of drought stress in grapevine by foliar-applied strigolactones. Plant Physiol. Biochem..

[81] Li X., Li Z., Shi Z., Wu T., Wu G., Sheng H., Zhou S., Wang L., Tang X., Liu J. (2026). Differences in physiological and ecological adaptations of *Ipomoea pes-caprae* in sandy beaches and coral islands. Glob. Ecol. Conserv..

[82] Zhang X., Ma C., Zhang L., Su M., Wang J., Zheng S., Zhang T. (2022). GR24-mediated enhancement of salt tolerance and roles of H_2_O_2_ and Ca^2+^ in regulating this enhancement in cucumber. J. Plant Physiol..

[83] Ma N., Hu C., Wan L., Hu Q., Xiong J., Zhang C. (2017). Strigolactones improve plant growth, photosynthesis, and alleviate oxidative stress under salinity in rapeseed. Front. Plant Sci..

[84] Zhang Y., van Dijk A.D.J., Scaffidi A., Flematti G.R., Hofmann M., Charnikhova T., Verstappen F., Hepworth J., van der Krol S., Leyser O. (2014). Rice cytochrome P450 MAX1 homologs catalyze distinct steps in strigolactone biosynthesis. Nat. Chem. Biol..

[85] Ma C., Bian C., Liu W., Sun Z., Xi X., Guo D., Liu X., Tian Y., Wang C., Zheng X. (2022). Strigolactone alleviates the salinity-alkalinity stress of *Malus hupehensis* seedlings. Front. Plant Sci..

[86] Zhang J., Feng N., Zheng D., Khan A., Du Y., Wang Y., Deng R., Wu J., Xiong J., Sun Z. (2024). Strigolactone Alleviates NaCl Stress by Regulating Antioxidant Capacity and Hormone Levels in Rice (*Oryza sativa* L.) Seedlings. Agriculture.

[87] Tai Z., Yin X., Fang Z., Shi G., Lou L., Cai Q. (2017). Exogenous GR24 Alleviates Cadmium Toxicity by Reducing Cadmium Uptake in Switchgrass (*Panicum virgatum*) Seedlings. Int. J. Environ. Res. Public Health.

[88] Nisa Z.U., Wang Y., Ali N., Chen C., Zhang X., Jin X., Yu L., Jing L., Chen C., Elansary H.O. (2023). Strigolactone signaling gene from soybean GmMAX2a enhances the drought and salt-alkaline resistance in *Arabidopsis* via regulating transcriptional profiles of stress-related genes. Funct. Integr. Genom..

[89] Jariani P., Sabokdast M., Rajabi F., Naghavi M.R., Dedicova B. (2025). Enhancing salinity tolerance in wheat: The role of synthetic Strigolactone (GR24) in modulating antioxidant enzyme activities, ion channels, and related gene expression in stress responses. Sci. Rep..

[90] Chi C., Xu X., Wang M., Zhang H., Fang P., Zhou J., Xia X., Shi K., Zhou Y., Yu J. (2021). Strigolactones positively regulate abscisic acid-dependent heat and cold tolerance in tomato. Hortic. Res..

[91] Li Q., Wang B., Yu H. (2025). New mechanism of strigolactone-regulated cold tolerance in tomato. New Phytol..

[92] Guo S., Wei X., Ma B., Ma Y., Yu Z., Li P. (2023). Foliar application of strigolactones improves the desiccation tolerance, grain yield and water use efficiency in dryland wheat through modulation of non-hydraulic root signals and antioxidant defense. Stress Biol..

[93] Mostofa M.G., Van Ha C., Rahman M.M., Nguyen K.H., Keya S.S., Watanabe Y., Itouga M., Hashem A., Abd Allah E.F., Fujita M. (2021). Strigolactones modulate cellular antioxidant defense mechanisms to mitigate arsenate toxicity in rice shoots. Antioxidants.

[94] Chi C., Chen X., Zhu C., Cao J., Li H., Fu Y., Qin G., Zhao J., Yu J., Zhou J. (2025). Strigolactones positively regulate HY5-dependent autophagy and the degradation of ubiquitinated proteins in response to cold stress in tomato. New Phytol..

[95] Wang D., Xi Z. (2022). Strigolactone agonists/antagonists for agricultural applications: New opportunities. Adv. Agrochem..

[96] Bari V.K., Nassar J.A., Aly R. (2021). CRISPR/Cas9 mediated mutagenesis of MORE AXILLARY GROWTH 1 in tomato confers resistance to root parasitic weed *Phelipanche aegyptiaca*. Sci. Rep..

[97] Janeeshma E., Habeeb H., Shackira A., Sinisha A., Mirshad P., Khoshru B., Henao S.G., Rani A., Verma D., Fathi A. (2024). Strigolactone and analogues: A new generation of plant hormones with multifactorial benefits in environmental sustainability. Environ. Exp. Bot..

[98] Li X., Lu J., Zhu X., Dong Y., Liu Y., Chu S., Xiong E., Zheng X., Jiao Y. (2023). AtMYBS1 negatively regulates heat tolerance by directly repressing the expression of MAX1 required for strigolactone biosynthesis in *Arabidopsis*. Plant Commun..

[99] Ali M., Wang Z., Guo Q., Wang Y., Cai Y., Du J., Pi E., Ding P., Shen J. (2026). Mapping plant cell-type-specific responses to environmental stresses. Trends Plant Sci..

[100] Butt H., Jamil M., Wang J.Y., Al-Babili S., Mahfouz M. (2018). Engineering plant architecture via CRISPR/Cas9-mediated alteration of strigolactone biosynthesis. BMC Plant Biol..

[101] Ban X., Qin L., Yan J., Wu J., Li Q., Su X., Hao Y., Hu Q., Kou L., Yan Z. (2025). Manipulation of a strigolactone transporter in tomato confers resistance to the parasitic weed broomrape. Innovation.

[102] Ali M., Kaderbek T., Khan M.A., Skalicky M., Brestic M., Elsabagh M., El Sabagh A. (2025). Biosynthesis and multifaceted roles of reactive species in plant defense mechanisms during environmental cues. Plant Stress.

[103] Jamil M., Mutinda S., Wang J.Y., Barminga D., Mwihaki A., Navangi L., Okiyo T.O., Patil R.H., Ngatia T., Mudavadi P. (2025). Evaluation of formulated strigolactone analogs for *Striga* management in Kenyan agriculture. J. Agric. Food Res..

[104] Kaniganti S., Palakolanu S.R., Thiombiano B., Damarasingh J., Bommineni P.R., Che P., Sharma K.K., Jones T., Bouwmeester H., Bhatnagar-Mathur P. (2025). Developing *Striga* resistance in sorghum by modulating host cues through CRISPR/Cas9 gene editing. Plant Cell Rep..

[105] Zhang C., Wang F., Jiao P., Liu J., Zhang H., Liu S., Guan S., Ma Y. (2024). The Overexpression of *Zea mays* Strigolactone Receptor Gene D14 Enhances Drought Resistance in *Arabidopsis thaliana* L.. Int. J. Mol. Sci..

[106] Kelly J.H., Tucker M.R., Brewer P.B. (2023). The Strigolactone Pathway Is a Target for Modifying Crop Shoot Architecture and Yield. Biology.

